# Pregnane X receptor-mediated liver growth: a controlled clinical trial in healthy volunteers and identification of the role of AKT–MTOR pathway in mouse

**DOI:** 10.1007/s00204-026-04526-5

**Published:** 2026-08-26

**Authors:** Piia Lassila, Mikko Karpale, Outi Kummu, Aaron Stahl, Aki Käräjämäki, Eija Pääkkö, Helen Sophie Hammer, Albert Braeuning, Oliver Pötz, Markus Templin, Janne Hukkanen, Jukka Hakkola

**Affiliations:** 1 Research Unit of Biomedicine and Internal Medicine, Pharmacology and Toxicology, Biocenter Oulu, Medical Research Center Oulu, Oulu University Hospital, University of Oulu, POB 5000, 90014 Oulu, Finland; 2 NMI Natural and Medical Sciences Institute at the University Tübingen, Reutlingen, Germany; 3 Department of Internal Medicine, Vaasa Central Hospital, Vaasa, Finland; 4 Department of Diagnostic Radiology, Oulu University Hospital, Oulu, Finland; 5 SIGNATOPE GmbH, Reutlingen, Germany; 6 Department of Food Safety, German Federal Institute for Risk Assessment (BfR), Berlin, Germany

**Keywords:** Liver hypertrophy, PXR, Rifampicin, PCN, AKT, MTOR

## Abstract

Pregnane X receptor (PXR) is a nuclear receptor acting as a master xenobiotic receptor for many exogenous chemicals. Activation of PXR has been linked to liver growth in mice, usually via interaction with Yes-associated protein (YAP), but this adaptive response has not been observed in humans. We investigated the effect of human PXR agonist, rifampicin, on liver size in a controlled clinical trial in healthy volunteers. Moreover, we developed a novel protocol to reveal mechanisms of PXR-mediated liver growth in mice. One-week rifampicin treatment caused a 2.7% mean increase in liver volume-to-body weight ratio without affecting liver fat content in human volunteers (n=16). Pxr^−/−^ mice were transduced with adenovirus carrying either murine PXR or green fluorescent protein and both groups were treated with pregnenolone−16α-carbonitrile (PCN) for 0 to 4 days. In this setup, activation of PXR with PCN significantly increased liver size in male mice already after 1-day PCN treatment and the liver growth was further enhanced after 4 days. No significant liver enlargement was observed in female mice. 4-day PCN treatment also improved glucose tolerance in the male mice. RNA-sequencing revealed upregulation of genes and pathways involved in proliferation. DigiWest protein profiling and immunoblotting revealed increased activity of AKT–mammalian target of rapamycin (MTOR) pathway in the male mice. In contrast, we did not observe YAP activation. Our results indicate that PXR activation induces liver growth and activates proliferative AKT–MTOR pathway in male mice and may also induce liver growth in humans.

**Supplementary Information:**

The online version contains supplementary material available at https://doi.org/10.1007/s00204-026-04526-5.

## Introduction

Liver has an unusual ability to regulate its size according to physiological needs and it is the only internal organ capable of regenerating its mass after liver injury or partial hepatectomy (Michalopoulos and Bhushan 2021). In rodents, liver size increases in response to several types of chemical exposures mediated by xenobiotic-sensing nuclear receptors such as pregnane X receptor (PXR), constitutive androstane receptor (CAR) and peroxisome proliferator receptor α (PPARα), but this adaptive response has not been observed in humans (Hall et al. 2012). Interestingly, these receptors appear to play a role also in liver regeneration and PXR is required for normal liver regeneration as indicated by insufficient liver growth in *Pxr*^*−/−*^ mice after partial hepatectomy (Dai et al. 2008).

PXR is a type II ligand-activated nuclear receptor classically defined as a master transcriptional regulator of several genes related to drug transport and drug metabolism (Rosenfeld et al. 2003). Additionally, PXR plays a role in regulating lipid, glucose and bile acid metabolism (Hukkanen et al. 2014; Hakkola et al. 2016). PXR activation by pregnenolone−16α-carbonitrile (PCN) (Jiang et al. 2019), dexamethasone (Jiao et al. 2020) and oleanolic acid (Song et al. 2024) has been linked to liver growth and liver regeneration. Mechanistically, PXR has been reported to promote liver growth and regeneration through a Yes-associated protein (YAP) mediated mechanism (Jiang et al. 2019).

In this investigation we show for the first time in a placebo-controlled clinical trial that PXR activator rifampicin induces liver hypertrophy also in humans. Furthermore, we developed a novel protocol to study the mechanisms of liver growth in mice by reintroducing PXR to the livers of *Pxr*^*−/−*^ mice and by treating the mice with a PXR ligand. With this novel model and targeted proteomics screening of relevant signaling pathways we revealed the AKT–MTOR pathway is strongly activated in the PXR-induced liver growth.

## Materials and methods

### Rifa-Stea clinical trial subjects and experimental setup

Healthy volunteers between the ages of 18- and 40-years old and body-mass index (BMI) between 18.5–25 kg/m^2^ were recruited for a clinical trial as described previously in more detail (Tolonen et al. 2023). Briefly, the exclusion criteria included significant diseases, any continuous medication, drugs and alcohol abuse, and pregnancy or breastfeeding. The trial had a placebo-controlled and randomized cross-over design where the participants received placebo or 600 mg rifampicin (Rimapen; Orion Corporation, Espoo, Finland) daily for a week with a minimum of 4-week washout period between treatments. MRI imaging to measure liver fat and liver size was performed on the following day (eighth day) after the last dose of either placebo or rifampicin. The radiologist responsible for the measurement of liver fat and size was blinded while the volunteers and trial site personnel were not blinded as rifampicin colors urine red rendering blinding impossible. The sequence (i.e., rifampicin or placebo period first) of the two arms was randomized. Plasma 4β-hydroxycholesterol (4βHC) as a marker of PXR-regulated cytochrome P450 3A (CYP3A) activity was measured on blood samples collected on the eighth day with the ultrahigh performance liquid chromatography coupled with high-resolution mass spectrometry method by Admescope (Oulu, Finland) (Hautajärvi et al. 2018) to monitor the compliance to rifampicin dosing (Diczfalusy et al. 2011). The subjects were asked to refrain from dietary and herbal supplements, vitamin supplements, anti-inflammatory drugs, alcohol and energy drinks during the trial arms.

The study procedures were performed in accordance with the ethical standards of the Declaration of Helsinki. The trial was approved by the Finnish Medicines Agency and the Ethics Committee of the Northern Ostrobothnia Hospital District (Oulu, Finland). Written informed consent was obtained from each volunteer. The subjects were compensated financially for participation. The sample size (n=16) was calculated with α = 0.05 and 1-β = 0.81 assuming that the true difference in the hepatic fat fraction between treatments is 1.20 percentage points and that the standard deviation of the difference in the response variable is 1.56. The trial was registered at ClinicalTrials.gov as NCT02329405.

### Liver size and hepatic fat fraction (triglycerides) measurements in human volunteers

MR imaging of the liver was performed by using Optima MR450w 1.5T (GE Healthcare Inc. Chicago, IL, USA) with commercially available sequences. The liver volume was measured on axial T2-weighted images covering the whole liver. The liver borders were traced manually by using a pencil tool in a workstation, avoiding the gall bladder and the central vascular areas. The obtained areas on each cross-sectional image were added together and multiplied by the section thickness and the gap between the slices, giving the volume in cubic millimeters. Hepatic fat fractions (triglycerides) were measured by using chemical shift imaging with IDEAL IQ sequence.

### Animals

The animal experiments were approved by the Project Authorisation Board, Finland (License number (ESAVI/8240/04.10.07/2017 and ESAVI/23252/2020) following EU legislation (Directive 2010/63/EU). *Pxr*^*−/−*^ mice in C57BL/6N background were bred in Laboratory Animal Centre, University of Oulu, Finland. *Pxr*^*−/−*^ mouse line was kindly provided by Professor Wen Xie, University of Pittsburgh School of Pharmacy Center for Pharmacogenetics, PA, USA. Mice were backcrossed to C57BL/6N strain for nine generations before they were utilized. During the experiment the mice were housed in individually ventilated cages with free access to regular chow and water with a controlled temperature of 22 °C and 40% humidity. A controlled 12h light cycle was maintained.

### Animal experiments

8-week-old C57BL/6N *Pxr*^*−/−*^ male mice, weighing 22-28g at the start of the experiment, and female mice, weighing 17-22g at the start of the experiment, were divided to groups (N=7/group) and transduced with either adenovirus carrying gene for murine PXR (Ad-GFP-mPXR, Cat. No. ADV-266039, Vector BioLabs, Malvern, PA, USA) or control adenovirus carrying gene for green fluorescent protein (Ad-GFP, Cat. No. 1060, Vector BioLabs, Malvern, PA, USA) via i.v. injection (~ 1×10^9^ of transduced units (TU)/mouse). Four days after the virus transduction (rest period) the mice were treated with 50 mg/kg PCN dissolved in DMSO/corn oil (Abcam, Cambridge, UK) i.p. once a day for 1, 2 or 4 days. Additionally, we performed a control experiment without PCN treatment (NO PCN) where male mice were maintained for 4 days after the ‘rest period’. Blood sample, as well as 0 min blood glucose measurement, were collected from saphenous vein after 12h fast and mice were given glucose 2 g/kg by oral gavage, and blood glucose was measured from the tail tip at 15, 30, 60, 90 and 120 min intervals. Mice were then sacrificed by CO_2_ inhalation and tissues were harvested and frozen in liquid nitrogen for RNA and protein isolation, or fixed in formalin solution for histology.

### RNA extraction and quantitative PCR (qPCR)

Total RNA was isolated from mice liver tissue using RNAzol (Sigma Aldrich, St. Louis, MO, USA) according to manufacturer’s protocol. 1 µg of RNA was used to synthesize cDNA using random hexamer primer (Roche, Basel, Switzerland) and reverse transcriptase enzyme (Thermo Fisher Scientific, Waltham, MA, USA) according to manufacturer’s protocol. cDNA samples were diluted 1:10 with H_2_O and quantitative PCR was performed in duplicates using PowerUP SYBR Green Master Mix (Thermo Fisher Scientific, Waltham, MA, USA) and QuantStudio5 instrument (Applied Biosystems). All results were normalized to mouse glyceraldehyde 3-phosphate dehydrogenase (*Gapdh)* and mouse TATA-binding protein (*Tbp)* mRNA using the comparative CT (ΔΔCT) method. The primer sequences are presented in Supplementary Table D.

### Protein isolation

Total protein isolation: Mouse liver samples (~ 100 mg) were homogenized with one stainless steel metal bead in 300 µl of lysis buffer ((20 mM Tris–Cl, 1 mM EDTA, 150 mM NaCl, 1% Triton-X100, 1 mM EGTA, 2.5 mM sodium pyrophosphate, 1 mM Na_3_VO_4_, 1 mM β-glycerophosphate, pH 7.5), 50 mM NaF, 1 mM DTT and supplemented with 1X Halt™ protease and phosphatase inhibitor cocktail (Thermo Fisher Scientific, Waltham, MA, USA)) using TissueLyzer LT (Qiagen, Hilden, Germany) with at 30^1/s^ shaking for 2 min. The lysate was centrifuged at 14,800 g for 20 min at +4°C and the supernatant was collected.

Cytoplasmic and nuclear protein fraction isolation: Mouse liver samples (~ 100 mg) were homogenized with one stainless steel metal bead in 4× volume of 0.1 M sodium-phosphate buffer pH7.4 with 1X Halt inhibitor cocktail. Collected supernatants were further diluted in 650 µl of 0.1 M sodium-phosphate buffer pH 7.4 with 1X Halt inhibitor cocktail and centrifuged at 14800g for 15 s at +4°C. Pellets were suspended in 400 µl of CNEA buffer (1 M HEPES pH 7.4, 2 M KCl, 0.25 M EDTA pH 8, 0.25 M EGTA pH 8, 1X Halt inhibitor cocktail) and incubated on ice for 15 min. 15µl of 10% Igepal was added and samples were vortexed for 15 s before centrifuging at 14800g for 30 s at +4°C. Supernatants were collected (= cytoplasmic proteins) and pellets suspended in 50 µl of CNEC buffer (1 M HEPES pH 7.4, 2.5 M NaCl, 0.25 M EDTA pH 8, 0.25 M EGTA pH 8, 1X Halt inhibitor cocktail). Samples were shaken for 15min at +4°C and centrifuged at 21400g for 10 min at +4°C. Supernatants were collected (= nuclear proteins). Total, cytoplasmic and nuclear fraction protein concentrations were measured using Bio-Rad (Bio-Rad, Hercules, CA, USA) protein assay (Bradford protein assay).

### Immunoblotting

Total liver proteins (20–50 µg) were separated using SDS-PAGE and transferred to nitrocellulose membrane with Turbo-Trans-Blot Turbo RT Mini Nitrocellulose Transfer Kit (Bio-Rad, Hercules, CA, USA) and Trans-Blot Turbo (Bio-Rad, Hercules, CA, USA) using Mixed MW-run protocol (1.3 A, 25 V, 7 min at RT) or user defined run protocol for YAP and p-YAP (1.3 A, 25 V, 15 min at RT). Membranes were blocked with Amersham ECL Prime Blocking Agent (GE Healthcare, Chigaco, IL, USA). Immunoblotting was performed using AKT, p-AKT (Ser473), S6, pS6 (Ser235/236), FOXO1, pFOXO1 (Ser256), YAP, p-YAP (Ser127) and β-actin primary antibodies (Supplementary Table E). All primary antibodies were diluted in Tris-buffered saline with 0.1% Tween 20 (0.1% TBST) and incubated overnight at +4°C. Secondary antibodies (Goat anti-mouse IRDye 680RD and Goat anti-rabbit IRDye 800CW) (Supplementary Table E) were diluted in 0.1% TBST and incubated RT for 1 h. Membranes were imaged with Odyssey Fc (LI-COR, Lincoln, NE, USA). Signals of primary antibodies were normalized to β-actin signal except for cytoplasmic and nuclear YAP fractions, which were normalized to total protein signal on membrane (Supplementary Table F).

### Immunohistochemistry

Histological changes in the mice livers were assessed by staining the liver samples with rabbit monoclonal Ki-67 and mouse monoclonal β-catenin (Supplementary Table E). Mouse liver samples were fixed in 10% formalin solution and embedded in paraffin and cut into 4 µm thick sections. Samples were heated in either citrate buffer pH 6 (β-catenin) at 800 W for 2min followed by 300 W for 10 min or tris–EDTA pH 9 at 800 W for 2 min followed by 300 W for 15 min before performing the staining protocol using Dako REAL EnVision Detection System kit (Agilent Technologies, Santa Clara, CA, USA). Samples were blocked for 5 min and primary antibodies (Ki-67 in 1:400 dilution and β-catenin in 1:200 dilution) were incubated for 1 h followed by secondary antibody incubation for 30 min. 3,3'-diaminobenzidine tetrahydrochloride (DAB) was used as a chromagen and incubated for 4min.

### Counting Ki-67 positive cells

The number of Ki-67 positive cells were counted from one 10× magnified area from stained slides on ImageJ (RRID:SCR_003070). After importing the image file to ImageJ, the file was converted from RBG Color to 16-bit greyscale image. Next, the image was converted to binary form to count the cells. After conversion, the threshold was adjusted until only the stained cells were selected. ‘Analyze Particles’-program was used to analyze and filter selected stained cells based on size (150-1000px^2^) and roundness (0.3–1.00).

### DigiWest method

DigiWest was performed as published (Treindl et al. 2016) using 12 µg of cellular protein. In brief, the NuPAGE system (Life Technologies) was used for gel electrophoresis and blotting onto PVDF membranes. Proteins were biotinylated on the membrane using NHS-PEG12-Biotin (50 µM) in phosphate-buffered saline containing 0.1% Tween-20 (PBST) for 1 h. Sample lanes were cut into 96 strips (0.5 mm each) and placed in one well of a 96-well plate before adding 10 µl elution buffer (8 M urea, 1% Triton-X100 in 100 mM Tris–HCl pH 9.5). Each strip/protein fraction was incubated with 1 distinct Neutravidin-coated MagPlex bead population (Luminex, Austin, TX, USA). Coupling was performed over-night and non-bound binding sites were blocked with 500 µM deactivated NHS-PEG12-Biotin for 1 h. By pooling all 96 protein-loaded bead populations, the original sample lane was reconstituted.

5 µl aliquots of bead mix were added to 96-well plates containing 50 µl assay buffer (Blocking Reagent for ELISA (Roche) supplemented with 0.2% milk powder, 0.05% Tween-20 and 0.02% sodium azide). Upon discarding of the assay buffer, 30 µl of primary antibody (diluted in assay buffer) was added per well. After overnight incubation at 15°C, the bead-mixes were washed twice with PBST and species-specific PE-labelled (Phycoerythrin) secondary antibodies (Dianova, Hamburg, Germany) were added for 1 h at 23°C. Beads were washed twice with PBST before readout on a Luminex FlexMAP 3D instrument.

109 primary antibodies (Supplementary Table A) were selected from a collection of > 1 500 available antibodies, all of which are performance-evaluated and routinely used in DigiWest. Peak integration was performed using an Excel-based analysis tool. Signal intensities (AFI = accumulated fluorescent intensity) were normalized (Supplementary Table B) to total protein amount loaded onto the beads. A total of 119 peaks were identified and yielding reliable signals (AFI > 50).

### CYP protein quantification by immunoaffinity-LC-MS/MS

Protein quantification was performed using a targeted proteomics approach, as previously described(Weiß et al. 2018). In brief, liver tissues were homogenized using a ball mill and incubated in lysis buffer (0.1% Triton X-100, 150 mM NaCl, 50 mM Tris, pH 7.4; phosphatase (Sigma Aldrich, St. Louis, MO, USA) and protease inhibitors (Roche, Basel, Switzerland)) at room temperature for 1 h. After centrifugation (3000×g, 5 min, 4 °C), supernatants were collected, and protein concentrations measured using a BCA assay (Thermo Fisher Scientific, Waltham, MA, USA). 150 µg of protein was subjected to proteolysis with trypsin (Pierce Trypsin Protease, MS grade; Thermo Scientific, Waltham, MA, USA). Surrogate peptides and internal standard peptides were precipitated with triple X proteomics (TXP) antibodies. Enriched peptides were separated in a 10-min nano LC-method and detected by parallel reaction monitoring (UltiMate 3000 RSLCnano and QExactive Plus, Thermo Fisher Scientific, Waltham, MA, USA) as described previously (Hammer et al. 2021). Raw LC–MS data were processed using TraceFinder 4.1 (Thermo Fisher Scientific, Waltham, MA, USA) and peptide amounts were calculated by the ratio between signal areas of endogenous peptides and isotopically labelled standards. Protein amounts were normalized and expressed as fmol/µg of extracted total protein. Samples that exceeded either fell under the lowest limit of detection or exceeded the upper limit of detection have been included in the data presented and given values that represent the lowest and highest limits of detection.

### Microsphere‑based array panel for β‑catenin profiling

Microsphere-based arrays were used to detect distinct molecular forms of β-catenin and analyze protein–protein interactions, as described previously (Luckert et al. 2011). In brief, the bead-based multiplex system employs color-coded beads each coated with a distinct capture antibody: pan-β-catenin capture antibody (R&D Systems, Minneapolis, MN, USA) and phospho-specific antibodies (Cell Signaling Technology, Danvers, MA, USA) targeting various phosphorylation sites (non-pSer33/Ser37/Thr41, pSer33/Ser37/Thr41, and pSer45) to differentiate β-catenin phosphorylation states. Recombinant GFP-tagged proteins—ECT, ICAT, and TCF4—were used as bait to capture free, active β-catenin as described previously (Groll et al. 2015). Anti-ms E-cadherin (R&D Systems, Minneapolis, MN, USA) was used as capture for the E-cadherin—β-catenin complex. Final detection step is performed by using a C-terminal-specific β-catenin antibody (BD Biosciences).

Samples were prepared as follows, liver tissues were homogenized using a ball mill and incubated in lysis buffer (0.1% Triton X-100, 150 mM NaCl, 50 mM Tris, pH 7.4; phosphatase (Sigma Aldrich, St. Louis, MO, USA) and protease inhibitors (Roche, Basel, Switzerland)) at room temperature for 1 h. After centrifugation (3000×g, 5 min, 4 °C), supernatants were collected, and protein concentrations measured using a BCA assay (Thermo Fisher Scientific, Waltham, MA, USA). Twenty micrograms of protein were diluted in assay buffer and incubated with capture beads overnight at 8°C and 600 rpm on a plate shaker. Microspheres were washed three times with 100 µl PBS, 0.1% Tween on a plate magnet. β-catenin antibody (BD Biosciences, Franklin Lakes, NJ, USA) was incubated with microspheres for another 60 min at RT and 600 rpm on a plate shaker followed by three additional washing steps. Finally, mouse-specific PE-labelled (Phycoerythrin) secondary antibody (Dianova, Hamburg, Germany) was added for 45 min at 23°C. Beads were washed twice with PBST before readout on a Luminex FlexMAP 3D instrument (Luminex, Austin, TX, USA). Protein extracts from HEK293 treated with 20 µM GSK3β-inhibitor SB216763 served as positive control.

### RNA-sequencing and pathway analyses

Livers from 1- and 4-day PCN-treated mice were transcriptionally profiled with RNA sequencing. Eight animals from each experiment were randomly selected (4 from control group and 4 from mPXR-group). Total liver RNA was DNAse treated with RNAse-free DNAse set (Qiagen, Hilden, Germany) together with RNeasy MinElute Cleanup kit (Qiagen, Hilden, Germany). For 1-day PCN treatment samples, RNA quantity, integrity and purity was determined with Agilent 5400. Messenger-RNA was purified from total RNA using poly-T oligo-attached magnetic beads, fragmented and cDNA libraries were prepared. Sequencing was done with NovaSeq 6000 platform (Illumina). For 4-day PCN-treated samples, RNA quality was measured using Advanced analytical fragment analyzer and sample concentration was measured with Qubit (Life Technologies). cDNA-library was prepared using Illumina Stranded Total RNA kit (Illumina). Sequencing was performed using Illumina NovaSeq 6000 platform.

RNAseq raw data was analyzed using web-based Chipster (RRID:SCR_010939) software. 150 bp or 100 bp paired-end reads were aligned to mouse Ensembl reference genome (Mus_musculus.GRCm38.95) with HISAT2 (RRID:SCR_015530) and reads per gene were counted with HTSeq (RRID:SCR_005514). Differentially expressed genes were determined with Deseq2 (RRID:SCR_015687) using Benjamini–Hochberg corrected adjusted *p*-value cutoff of < 0.05. Full data can be accessed at GSE297681 and GSE297893.

Data was analyzed using Ingenuity Pathway analysis program (IPA) (Krämer et al. 2014) (Qiagen, Hilden, Germany) to determine canonical pathways associated with liver growth in 1- and 4-day PCN-treated male mice. Fischer’s exact test [a-Log(P-value) <0.05] was used as a threshold to calculate the significance of results.

### Statistical analysis

Data are presented as means ± SD. The statistical analysis of human clinical trial and animal studies were carried out using GraphPad Prism (GraphPad Software, La Jolla, CA, USA; RRID:SCR_002798). Differences between two groups were compared by using parametric two-tailed single or multiple Student’s t-test (paired for clinical trials and unpaired for animal studies), *p< 0.05 was considered statistically significant.

## Results

### Rifampicin increased liver size in healthy human volunteers

To investigate the effect of PXR activator rifampicin on the hepatic fat fraction and size of human liver, we performed a randomized, placebo-controlled, cross-over clinical trial where healthy volunteers (n=16) were treated orally with 600 mg of rifampicin once a day for one week. One participant dropped out from the trial due to a health problem unrelated to the study procedures after the first arm and a replacement volunteer was recruited. MRI measurements were performed for all 16 volunteers, but the IDEAL IQ sequence for the measurement of hepatic fat fraction was not successful for one volunteer recruited due to a technical problem. Thus, the number of volunteers for the paired measurements of the hepatic fat fraction and liver volume measurements were n=15 and n=16 (5 women and 11 men), respectively.

**Fig. 1:**
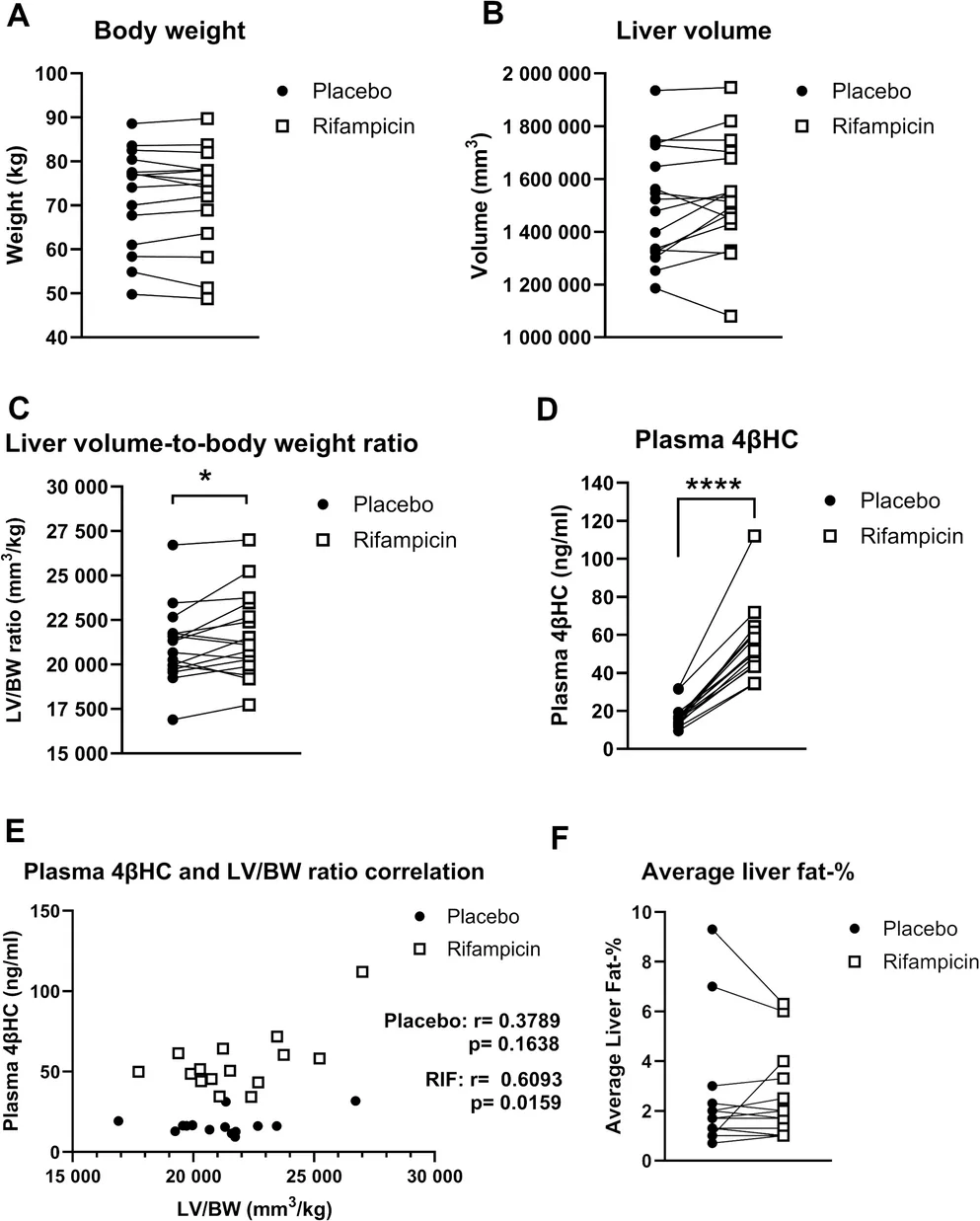
In a placebo-controlled, randomized, cross-over trial, one-week rifampicin treatment did not have effect on **A** body weight or **B** liver volume. Rifampicin did increase **C** liver volume-to-body weight ratio by 2.7% as well as increased **D** plasma 4βHC. **E** Plasma 4βHC correlated positively with liver volume-to-body weight ratio (LV/BW) in rifampicin arm. **F** Rifampicin did not influence average liver fat%. *p<0.05; ****p<0.0001

Rifampicin treatment did not affect body weight (placebo: 71.9kg ± 11kg, rifampicin: 71.8kg ± 11 kg but caused a mean 2.6% increase in the liver volume although the result was not significant (Fig. 1A, B). However, comparing the ratio of liver volume to body weight measured during the same day as MRI imaging, rifampicin treatment caused a significant 2.7% (*p*=0.042) mean increase in the liver volume-to-body weight ratio (Fig. 1C). Rifampicin caused a significant increase in mean plasma 4β-hydroxycholesterol (4βHC) levels (placebo: 17.13ng/ml ± 6.36ng/ml, rifampicin: 55.37ng/ml ± 18.9ng/ml) that reflects CYP3A activity and thus PXR activation (Fig. 1D). Plasma 4βHC was elevated in all volunteers indicating adherence to rifampicin dosing. There was a positive correlation between the plasma 4βHC and liver volume-to-body weight ratio after the rifampicin treatment but not in the placebo arm (Fig. 1E).

Rifampicin did not affect the average liver fat content (placebo: 2.45% ± 1.76% and rifampicin: 2.49% ± 2.32%) (Fig. 1F). Average liver fat-% did not correlate with liver volume or liver volume-to-body weight ratio (LV/BW ratio) in the placebo or rifampicin arms (Supplementary Fig. 1A,B).

### Activation of re-expressed mPXR by PCN significantly increased liver size in male *Pxr*^*-/-*^ mice

**Fig. 2:**
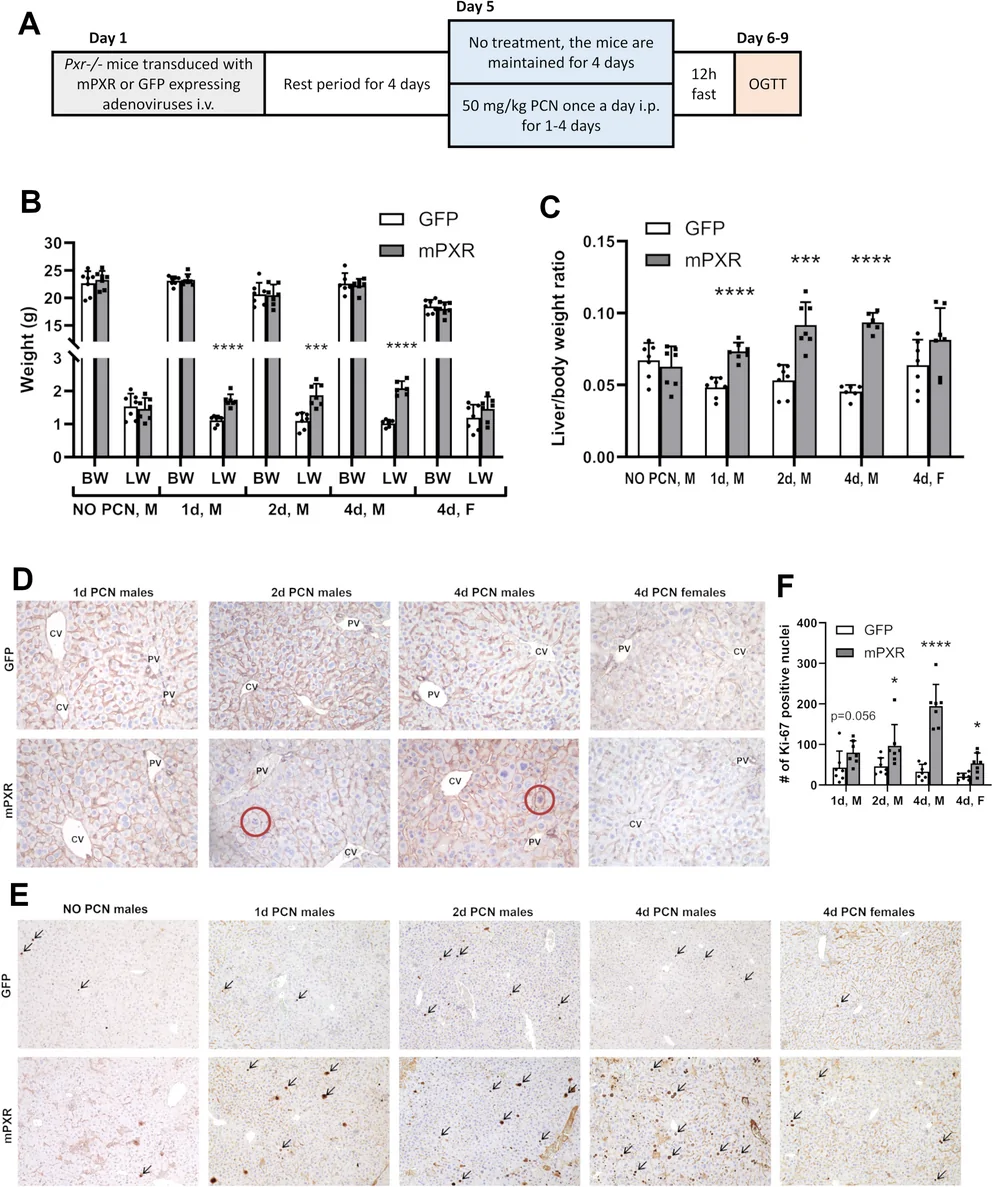
**A** Graphical representation of the animal experiments. Activation of re-expressed mPXR by PCN significantly increased **B** liver size and **C** liver-to-body weight ratio in male mice. **D** Beta-catenin staining of the mouse livers. Representative cells undergoing mitosis marked with red circles. **E** Ki-67 staining of the mouse livers. Ki-67 positive nuclei are marked with black arrow. **F** Quantitation of Ki-67 positive nuclei shows that activation of mPXR by PCN significantly increased the amount of Ki-67 positive cells in 2- and 4-day PCN-treated males as well as in females. BW=body weight, LW=liver weight, d=days, M=male, F=female. Results are presented as mean ± SD (n=7), *p<0.05; ***p<0.001; ****p<0.0001

In the next step, we proceeded to study mechanisms of the PXR-mediated liver growth with a novel experimental setup in mice. We re-expressed murine PXR via adenovirus delivery in the livers of adult male C57BL/6N *Pxr*^*−/−*^ mice. The control mice were treated with GFP expressing control adenovirus and consequently all the mice were treated with 50 mg/kg PCN i.p. once a day for 1, 2 or 4 days (Fig. 2A). To reveal possible sex differences in the phenomenon, we also subjected female C57BL/6N *Pxr*^*−/−*^ mice to the similar treatment, however, with 4-day PCN treatment only. We also investigated the effect of mPXR re-expression alone without PCN treatment in male mice (NO PCN). After the treatments, the mice were fasted for 12h and subjected to an oral glucose tolerance test (OGTT). Finally, the mice were sacrificed, and tissues were collected for analysis (Fig. 2A).

Significant liver growth was observed after mPXR re-expression and PCN treatment in male mice in all time points. After 1-, 2- or 4-days of PCN treatment liver size increased 1.7-fold, 1.9-fold and 2.0-fold, respectively (Fig. 2B). At the same time, the body weights were not affected and the liver to body weight ratio increased 1.5-fold, 1.7-fold and 2.0-fold, respectively (Fig. 2C). In contrast, no significant effect of combined mPXR re-expression and PCN treatment on liver size was observed in female mice (Fig. 2B, C). It should be noted that *Cyp3a11* expression was similarly induced by PXR re-expression and PCN treatment in the livers of both sexes indicating that the observed sex difference in liver growth was not due to any technical issue or difference in PXR activation between males and females (Supplementary Fig. 2). Furthermore, mPXR re-expression alone without PCN treatment had no effect on liver weight (Fig. 2B, C).

### mPXR activation-induced liver growth involves both hepatocyte hypertrophy and increased proliferation

To study microscopic changes in the liver structure, histological samples from the largest lobule of the mouse livers were fixed in formalin, 4 µm cross-sections were made and stained with an antibody against β-catenin to visualize cell membranes. In the male mice, the increased liver size in the mPXR mice was associated with hypertrophy of hepatocytes that could be observed throughout the liver lobules but especially in the pericentral area. The hypertrophy could be observed at all time points, but it was the most prominent at the last time-point after 4-day PCN treatment. In contrast, no obvious hepatocyte hypertrophy was observed in the mPXR treated female mice livers after 4-days PCN treatment (Fig. 2D). In addition, increased nucleus size could be observed in the livers of mPXR expressing, PCN-treated male mice livers in all the time points, however, most clearly in the 4-day PCN-treated mice (Fig. 2D). The increase in nucleus size may be related to increased DNA content per nucleus. In contrast, mPXR expression and 4-day PCN treatment did not affect nucleus size in the livers of female mice. Interestingly, a few hepatocytes were going through mitosis both in the PXR-expressing, PCN-treated mice liver, but also in control GFP, PCN-treated mice liver, examples are pointed out in Fig. 2D.

Next, we stained the liver sections with a Ki-67 antibody to detect proliferating liver cells. PXR dependent liver growth was associated with increased amount of Ki-67 positive nuclei that were detected in both male and female mice, and this was especially clear in male mice after 4-day PCN treatment (Fig. 2E, F).

HE-staining of the livers revealed mild hepatosteatosis in the mPXR re-expressing and 4-day PCN-treated mice (Supplementary Fig. 3.).

### Activation of re-expressed mPXR by PCN improved glucose tolerance in male mice

**Fig. 3:**
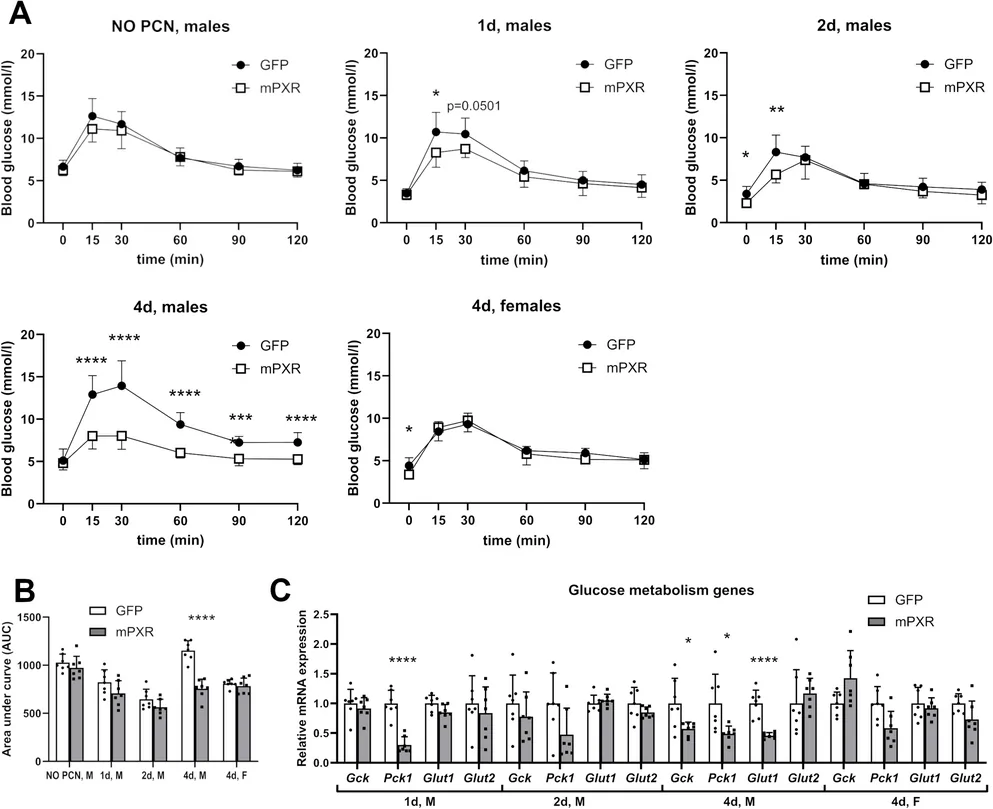
**A** Blood glucose levels during OGTT in mPXR re-expressing male mice without PCN treatment, and after 1-, 2- or 4-day PCN treatment, and in mPXR re-expressing female mice after 4-day PCN treatment. **B** AUC values of OGTT results from panel A. **C** Relative mRNA expression of glucose metabolism genes in the livers of mPXR re-expressing PCN-treated mice. d=days, M=male, F=female. Results are presented as mean ± SD (n=7), *p<0.05; **p<0.01; ***p<0.001; ****p<0.0001

1- and 2-day treatment with PCN improved glucose tolerance during the first 15 min of OGTT in the male mice (Fig. 3A). Remarkably, the 4-day treatment with PCN improved glucose tolerance very efficiently at all time points in the male mice and the area under curve (AUC) decreased 34% (Fig. 3A, B). No effect on glucose tolerance was observed in the female mice (Fig. 3A, B), nor in the male mice with mPXR re-expression only without PCN treatment (Fig. 3A, B).

Expression of several glucose metabolism-related genes previously described to be regulated by PXR were measured from the livers with qPCR. Glucokinase (*Gck),* phosphoenolpyruvate carboxykinase 1 *(Pck1)* and glucose transporter 1 *(Glut1)* were significantly downregulated in males after 4-day PCN treatment. Of these only *Pck1* was significantly repressed already after 1-day PCN treatment. After 2-day PCN treatment *Pck1* tended to be downregulated, but the effect was not statistically significant (Fig. 3C).

### RNA-sequencing analysis reveals increased xenobiotic metabolism and cell cycle progression

**Fig. 4:**
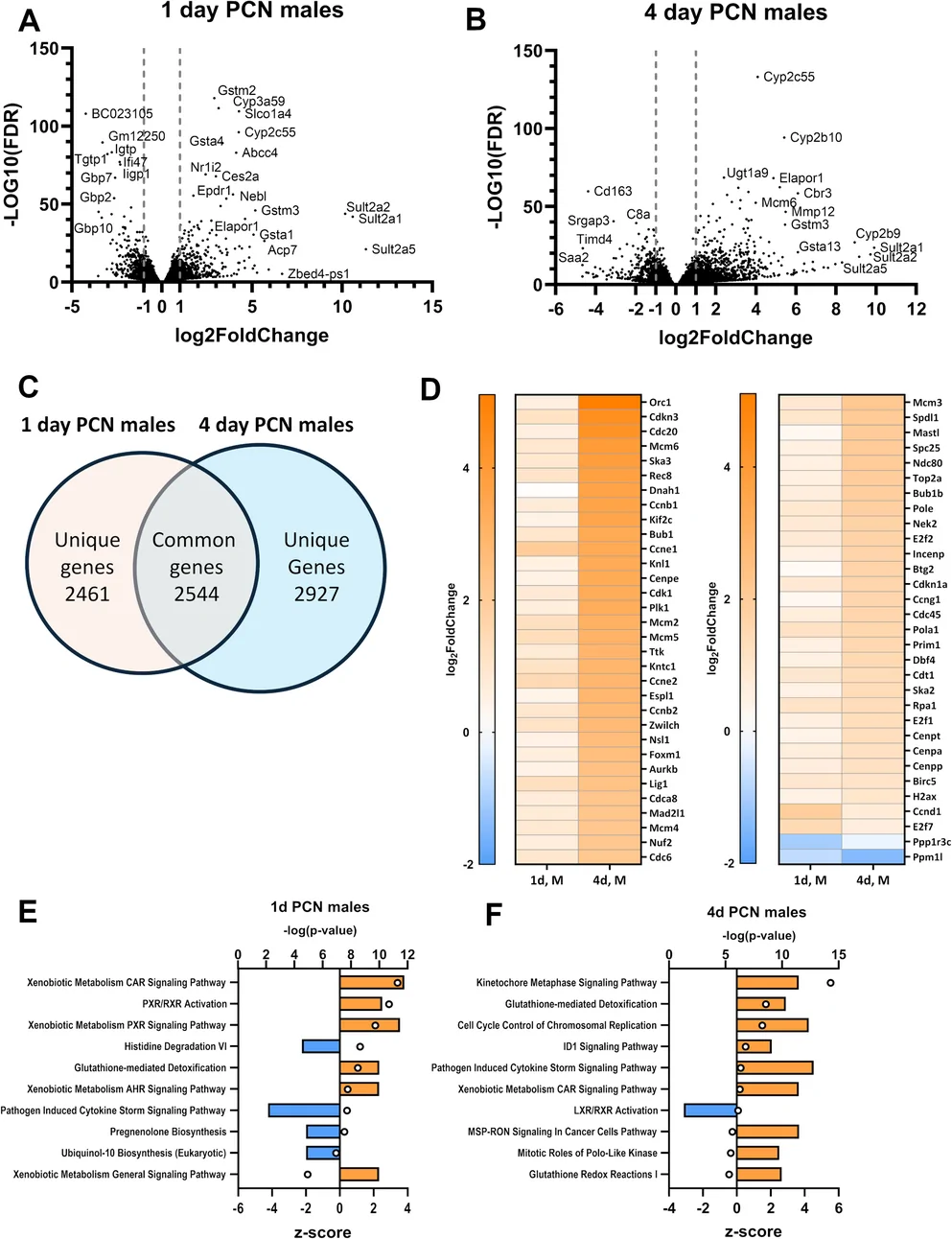
RNA-sequencing of the liver samples from mPXR re-expressing male mice after 1- or 4-day PCN treatment. Volcano plots of differentially expressed genes (DEGs) (*p*<0.05) from **A** 1-day PCN treatment, and **B** 4-day PCN treatment. Dashed lines represent log_2_FoldChange values of 1 and − 1. **C** 1- and 4-day treated mice shared 2544 differentially expressed genes (*p*<0.05) and had 2461 and 2927 unique genes, respectively. **D** Several DEGs associated with proliferation and cell cycle progression were upregulated in both time points. **E**, **F** Ingenuity Pathway Analysis (IPA) revealed that xenobiotic metabolism associated pathways were activated in 1-day PCN-treated males whereas pathways associated with proliferation and cell cycle were activated in 4-day PCN-treated males, based on z-score and -log(*p*-values). -log(*p*-value)<1.3 and z-score >|2| were considered significant

To further identify mechanism of PXR-induced liver growth, a set of livers samples from the male *Pxr*^*−/−*^ mice re-expressing mPXR, and the corresponding GFP controls, treated with PCN for 1 or 4 days were subjected to RNA-sequencing. Altogether, RNAseq revealed 5005 differentially expressed genes (DEGs) (2566 up- and 2439 downregulated) in 1-day PCN treatment and 5471 DEGs (3031 up- and 2440 downregulated) in 4-day PCN treatment (Fig. 4A, B). 2544 DEGs were found in both time points (1320 up- and 1224 downregulated in 1d males, and 1414 up- and 1130 downregulates in 4d males) (Fig. 4C). Expectedly, and in accordance with the function of PXR in regulation of detoxification, many genes involved in drug metabolism, for example, cytochrome P450 2c55 *(Cyp2c55)*, sulfotransferase 2a1 (*Sult2a1), Sult2a2, Sult2a5 and* glutathione S-transferase mu 3 (*Gstm3),* were highly induced at both time points (Fig. 4A, B). Genes associated with immune response (interferon gamma induced GTPase (*Igtp),* T-cell specific GTPase 1 *(Tgtp1),* interferon inducible GTPase 1 *(Iigp1),* interferon-induced guanylate-binding protein 2 *(Gbp2)* and *Gbp7* were inhibited after 1-day PCN treatment (Fig. 4A). Interestingly, cluster of differentiation 163 (*Cd163),* a gene expressed selectively in monocyte/macrophage cell lineage (including Kupffer cells) was among the most strongly repressed genes after 4-day PCN treatment (Fig. 4B).

Ingenuity Pathway Analysis revealed that after 1-day PCN treatment the top upregulated pathways were dominated by several pathways related to xenobiotic metabolism and detoxification such as xenobiotic metabolism related to PXR, constitutive androstane receptor (CAR) or aryl hydrocarbon receptor (AHR) pathways, and glutathione-mediated detoxification (Fig. 4E). Some of these xenobiotic detoxification pathways remain among the top pathways also after 4-day PCN treatment, however, at this time point the top pathways are predominantly associated with cell cycle progression and growth, i.e., kinetochore metaphase signaling pathway, cell cycle control of chromosomal replication, mitotic roles of polo-like kinase, MSP-RON signaling and ID1 signaling (Fig. 4F). In accordance, genes related to the pathways mentioned above were more highly induced after 4-day than 1-day PCN treatment (Fig. 4D).

Among the 10 most significantly affected pathways, LXR/RXR activation was the only one predicted to be downregulated (negative z-score) after 4-day PCN treatment. Interestingly, repression of LXR function has been reported to be necessary for liver regeneration after partial hepatectomy (Lo Sasso et al. 2010). Pathogen induced cytokine storm-signaling pathway was predicted to be repressed after 1-day but activated after 4-day PCN treatment.

### Characterization of pathways activated in liver growth by targeted DigiWest proteomics reveals activation of AKT–MTOR signaling

To gain further insight into the mechanism of PXR-mediated liver growth we next performed targeted proteomics and phosphoproteomics with the DigiWest-method (Treindl et al. 2016). A set of samples from the 1- and 4-day PCN-treated animals, which were also used for sequencing, were selected for DigiWest analysis of 109 analytes (Supplementary Table A.).

As a reference for mPXR activation we included pan-specific antibodies for CYP2B and CYP3A in the DigiWest analysis. Expectedly, proteins immunoreactive with these antibodies were induced already after 1-day PCN treatment (3- and 7-fold, respectively) and the induction was further enhanced after 4-day treatment (6- and 9-fold respectively) (Supplementary Table C). Furthermore, absolute quantification IA-LC–MS analysis confirmed induction of CYP2B10 and CYP2C55 (Supplementary Fig. 4).

**Fig. 5:**
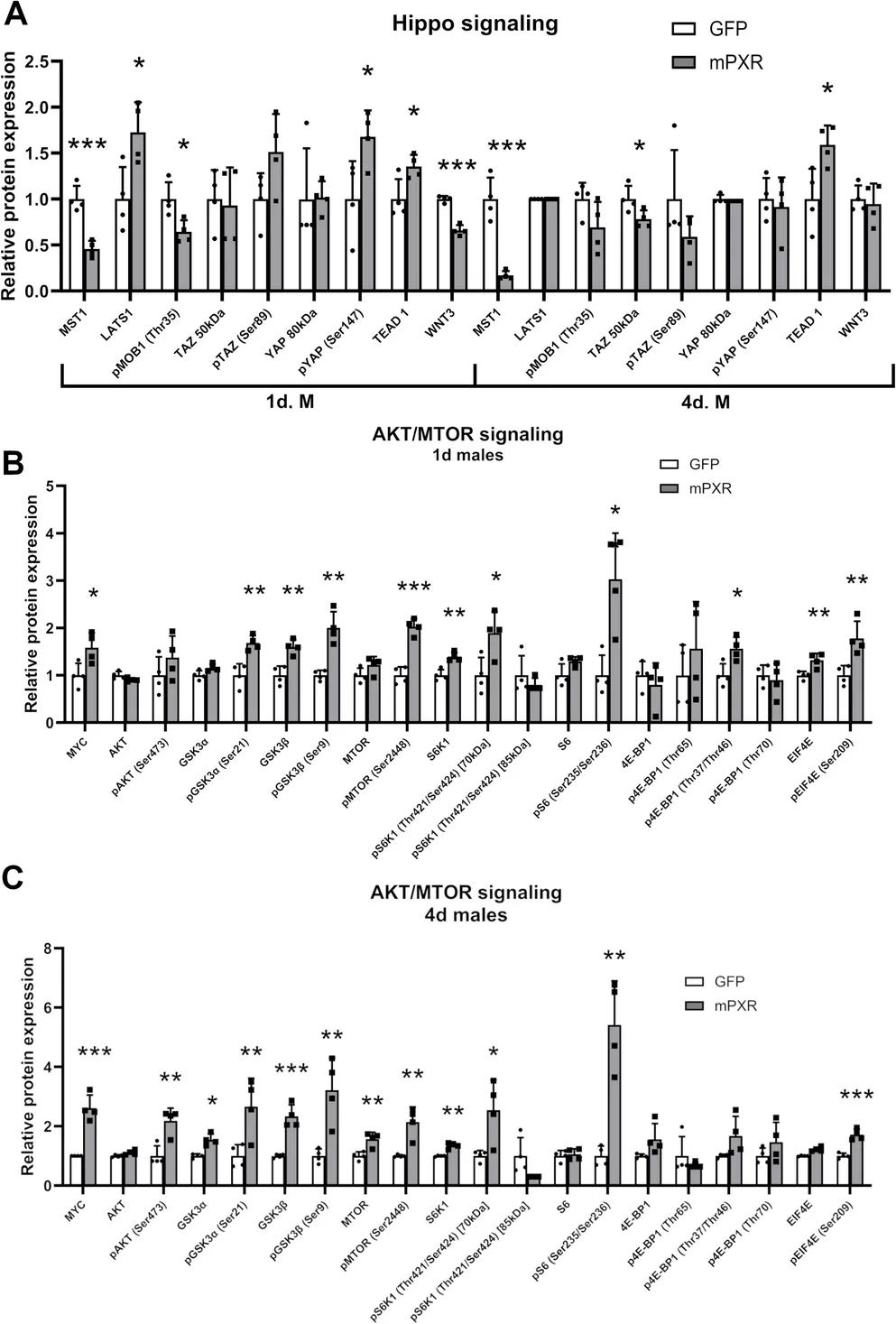
DigiWest analysis of liver samples from mPXR re-expressing and male mice after 1- or 4- day PCN treatment. **A** Hippo signaling pathway, and **B**, **C** AKT–MTOR signaling pathway. d=days, M=males. Results are presented as mean ± SD (n=7), *p<0.05; **p<0.01; ***p<0.001; ****p<0.0001

Next, we focused on pathways potentially involved in liver growth. The Hippo pathway is considered a key pathway in liver growth and regeneration. MST1 and MST2 are the mammalian homologs of Hippo kinase and thus central regulators of the Hippo pathway upstream of YAP. MST1 expression was efficiently down-regulated in the mouse livers re-expressing mPXR after 1-day PCN treatment and even further decreased after 4-day PCN treatment (Fig. 5A, Supplementary Table C). This could suggest that the Hippo pathway was switched off resulting in stabilization and nuclear localization of YAP and transactivation through TEAD1. However, protein levels of YAP as well as its homolog TAZ were not affected by PCN treatment at either timepoint. Furthermore, at the 1-day time point YAP phosphorylation (pS127) was increased and that of TAZ tended to be increased suggesting that the Hippo pathway would be active (Fig. 5A, Supplementary Fig. 6).

**Fig. 6:**
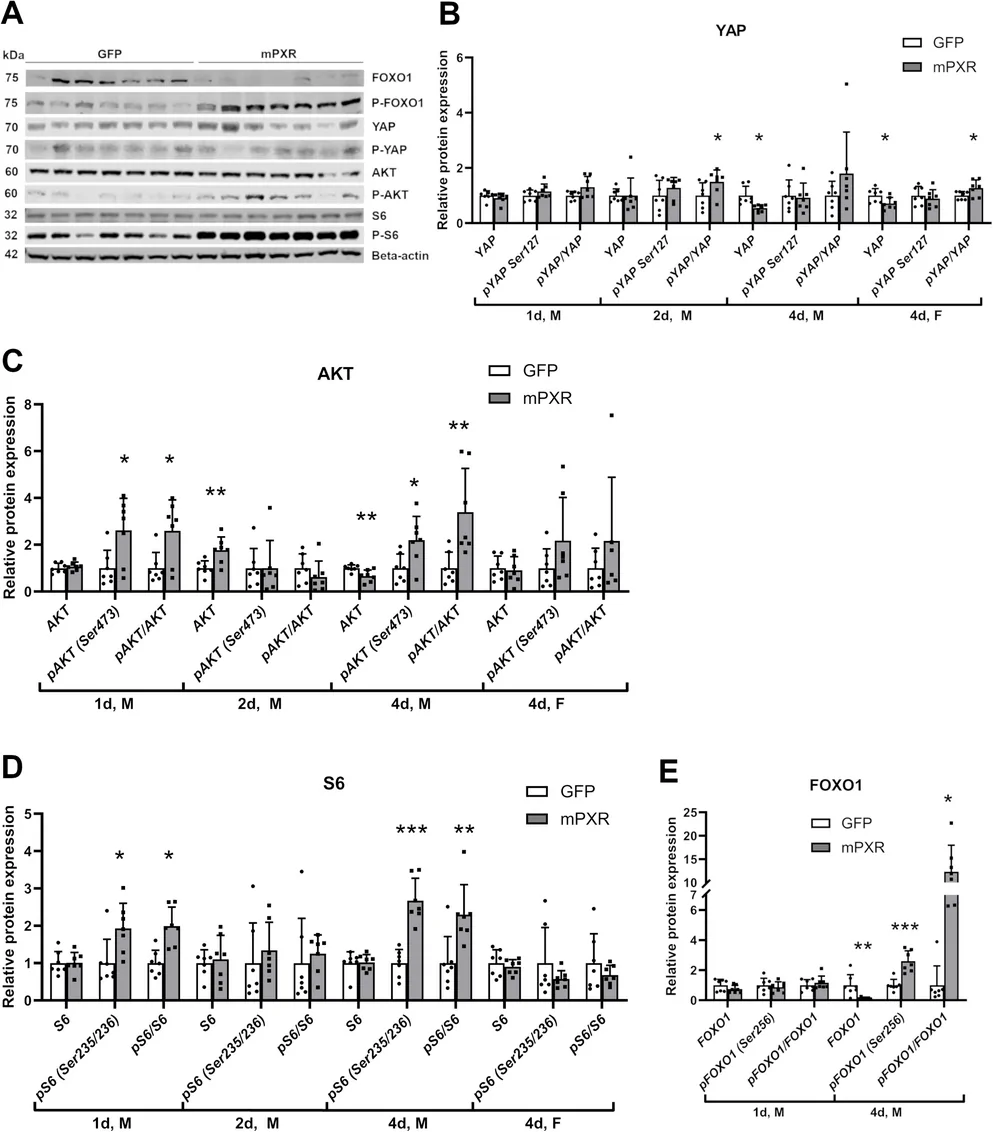
Protein expression analyses of the liver samples from mPXR re-expressing male and female mice after PCN treatment of indicated length. **A** Immunoblots of the 4-day PCN-treated male liver samples. Quantitation of immunoblots **B** YAP and pYAP, **C** AKT and pAKT, **D** S6 and pS6, **E** FOXO1 and pFOXO1. d=days, M=males, F=females. Results are presented as mean ± SD (n=7), *p<0.05; **p<0.01; ***p<0.001

To further characterize the activity of YAP we performed Western blotting for full sample sets at all time points and both sexes (Fig. 6A, B). YAP protein level was unaffected in the livers of male mice after 1- and 2-day PCN treatment but was decreased after 4-day treatment. Phospho-YAP and total-YAP ratio was increased significantly after 2-day PCN treatment and also in the female mice after 4-day PCN treatment (Fig. 6B). These results support the DigiWest results suggesting that Hippo pathway is “ON”, and therefore YAP is not promoting expression of its target genes.

**Fig. 7:**
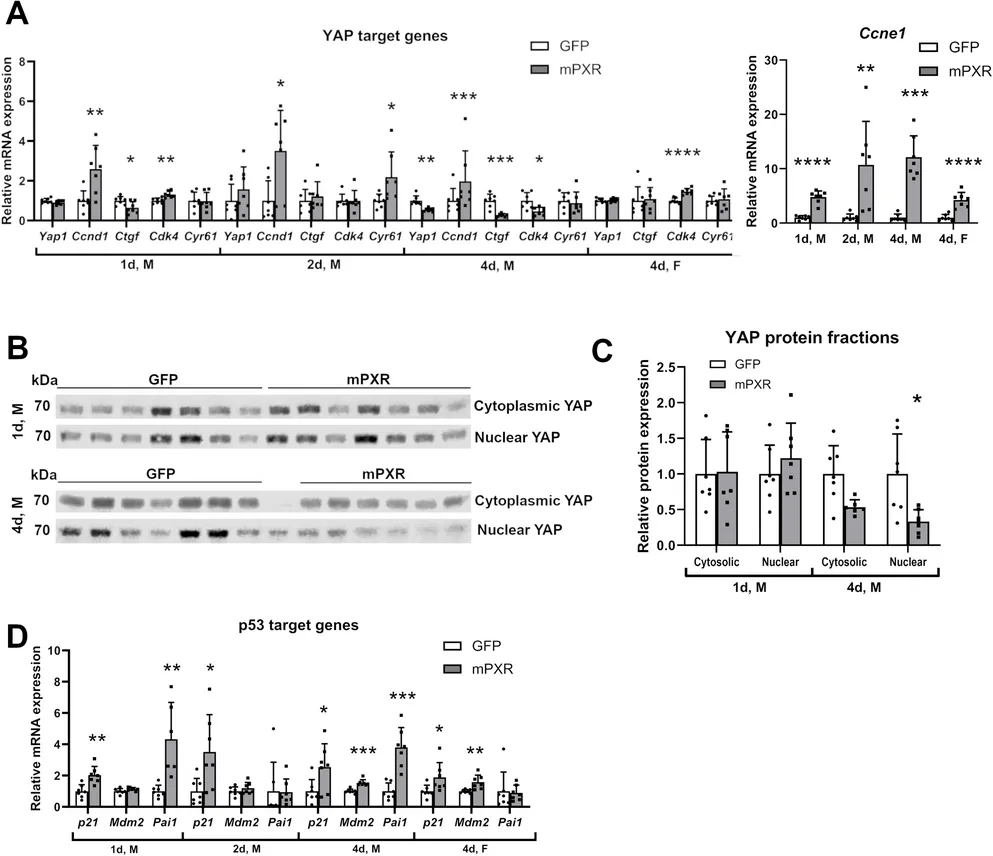
**A** Expression profile of YAP target genes in the liver samples of mPXR re-expressing male and female mice after PCN treatment of indicated length. **B** Immunoblotting of the cytoplasmic and nuclear fractions of YAP in in the livers of mPXR re-expressing male mice after 1- and 4-day PCN treatment. **C** Quantitation of the cytosolic and the nuclear YAP signal from panel B. In 4-day cytoplasmic samples only 6 samples were analyzed due to a technical error in one mPXR sample. **D** Expression profile of p53 target genes in the livers of mPXR re-expressing male and female mice after PCN treatment of indicated length. d=days, M=males, F=females. Results are presented as mean ± SD (n=7), *p<0.05; **p<0.01; ***p<0.001; ****p<0.0001

Next, we performed qPCR analysis for genes related to proliferation and recently described to be regulated by PCN either YAP dependently or independently based on the use of the YAP inhibitor verteporfin (Zhang et al. 2023). Verteporfin was reported to prevent PCN-mediated induction of connective tissue growth factor (*Ctgf)*, cysteine-rich angiogenic inducer 61 (*Cyr61, official name Ccn1)* and cyclin-dependent kinase 4 (*Cdk4)* but not that of cyclin E4 (*Cnne1)* and *Ccnd1* (Zhang et al. 2023). In our experiment, *Cnne1* and *Ccnd1* were induced by PCN treatment in male mice at all timepoints. *Ctgf* was repressed at the 1- and 4-day timepoints and no effect was detected in the 2-day timepoint. *Cdk4* was slightly induced after 1-day PCN treatment, no effect was detected after 2-day treatment, and after 4-day PCN treatment the gene was repressed. *Cyr61* was induced only after 2-day PCN treatment, and no effect was detected at other time points (Fig. 7A). Thus, the strong induction of *Cnne1* and *Ccnd1* and either lack of induction or inconsistent inductions of the genes dependent on YAP activity suggest no, or only minor, activation of YAP in the conditions studied.

Finally, to investigate potential YAP activation we performed immunoblotting of YAP in nuclear and cytosolic fractions from the livers of the male mice treated with PCN for 1 or 4 days (Fig. 7B, C). We did not detect any significant change in cytosolic or nuclear YAP after 1-day treatment with PCN while the amount of nuclear YAP was decreased after 4-day PCN treatment suggesting the lack of YAP activation.

Canonical Wnt-β-catenin signaling is a highly conserved pathway that regulates liver homeostasis. Aberrant signaling can lead to benign or malignant hepatobiliary diseases. β-catenin is the key effector of the pathway. In the absence of Wnt ligands, phosphorylated β-catenin is targeted for proteosomal degradation. in the presence of Wnt ligands, β-catenin becomes dephosphorylated and translocates to nucleus where it regulates the expression of its target genes (Perugorria et al. 2019). β-catenin level as well as the non-phosphorylated (stabilized) form of β-catenin were decreased after 4-day PCN treatment (Supplementary Table C) suggesting inactivity of WNT/β-catenin signaling. To further confirm the status of this pathway we performed microsophere-based β-catenin assays to investigate the status of transcriptionally active, phospho-/dephospho-forms and the total amount of β-catenin. No statistically significant differences were observed in the total β-catenin, the specific phospho-/dephospho-forms or transcriptionally active β-catenin (Supplementary Fig. 5).

**Fig. 8:**
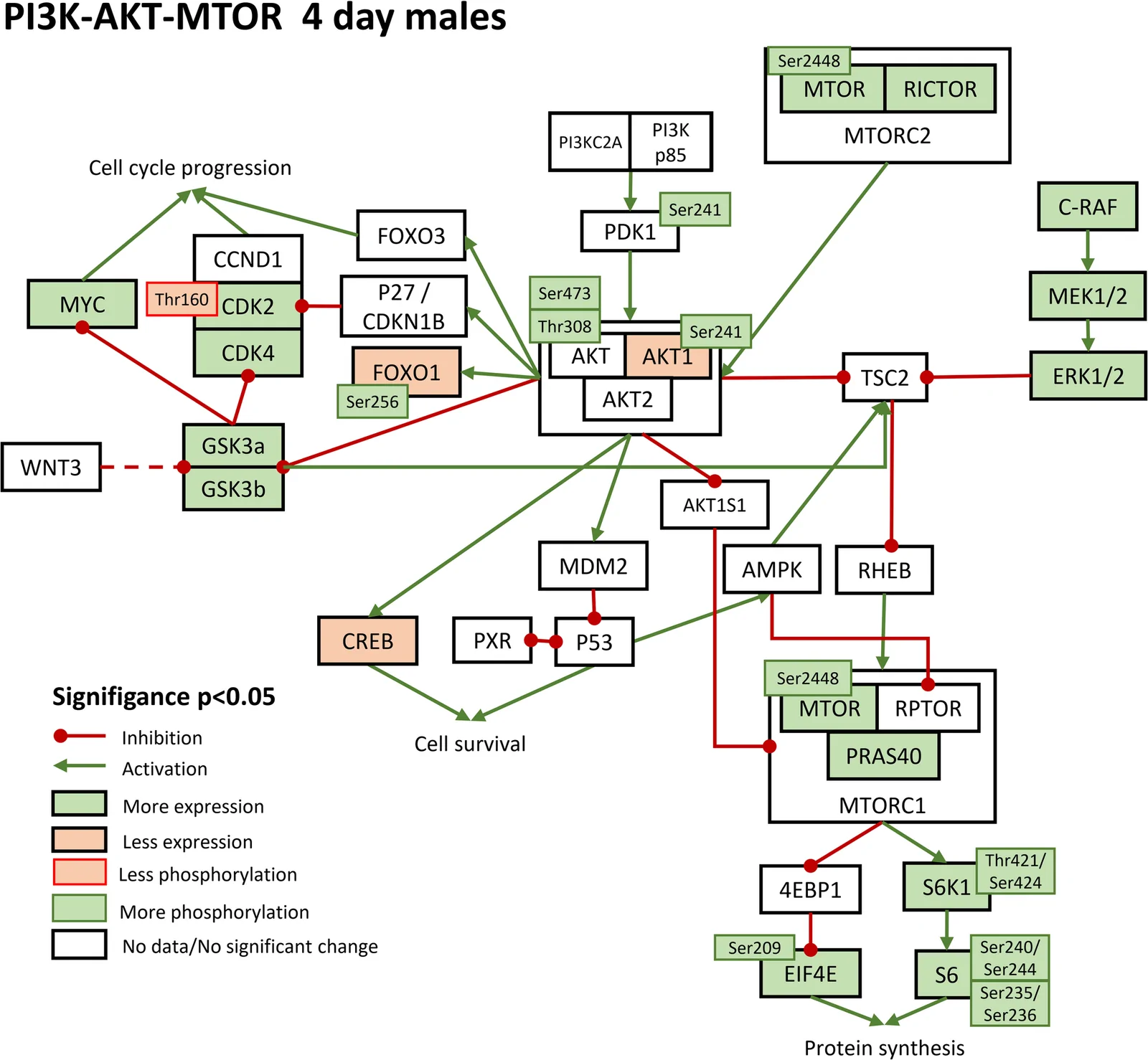
Graphical representation of the activation of PI3K–AKT–MTOR pathway in DigiWest and western blot protein profiling in the livers of mPXR re-expressing male mice livers after 4-day PCN treatment. Statistical significance level *p*<0.05

PI3K–AKT–MTOR pathway is an important regulator of cell cycle as well as regulator of glucose and fat metabolism, all functions affected during liver growth. In the DigiWest data, there was clear evidence of activation of the AKT–MTOR pathway after 4-day PCN treatment: AKT phosphorylation, MTOR protein level and phosphorylation as well as ribosomal protein S6 (S6) phosphorylation were all increased (Figs. 5C and 8). After 1-day PCN treatment, increased MTOR and S6 phosphorylations were detected (Fig. 5B, Supplementary Table C and Supplementary Fig. 7). We also performed immunoblotting for the whole sample set in different time points to further confirm the activation of this pathway. AKT and S6 phosphorylations were clearly increased both after 1 and 4-day PCN treatments (Fig. 6A, C, D).

AKT-mediated FOXO1 phosphorylation controls FOXO1 subcellular localization and consequently it’s function. AKT-mediated FOXO1 inhibition has been reported to be required for the liver regeneration (Pauta et al. 2016). We therefore measured FOXO1 status after 1- and 4-day PCN treatment by immunoblotting. At the 4-day time point FOXO1 phosphorylation was induced and the total FOXO1 level was decreased, suggesting inhibition of FOXO1 function (Fig. 6E.).

Tumor suppressor protein p53 and PXR have been described to have mutual inhibitory effect on each other. Furthermore, p53 and MTOR have opposite effect on liver regeneration but also share some common upstream regulation (Zhang et al. 2015). Therefore, we assessed the activity of p53 pathway. We did not observe significant changes in p53 protein levels (Supplementary Table C); however, p53 target genes cyclin dependent kinase inhibitor 1 (*p21)* and plasminogen activator inhibitor-1 (*Pai1)* were significantly induced after 1- and 4-day PCN treatments in males, suggesting an increased activity of p53 and potential inhibition of PXR activation-related growth (Fig. 7D).

## Discussion

Multiple mechanisms ensure that liver-to-body weight ratio is maintained as required by body homeostasis to maintain the “hepatostat” (Michalopoulos 2017; Michalopoulos and Bhushan 2021; Sarkar et al. 2023). In accordance, the liver has a unique regenerative capacity after partial hepatectomy or chemical-induced liver injury (Michalopoulos and Bhushan 2021). In some situations, however, the liver size may not follow the “hepatostat” principle. The liver size is increased during pregnancy unlinked to body size both in humans and rodents (Bartlett et al. 2021). Furthermore, liver hypertrophy is a well-known phenomenon in rodents, which is induced by exposure to chemicals activating xenobiotic -sensing nuclear receptors, such as PXR (Hall et al. 2012). However, the xenobiotic-induced liver hypertrophy has been assumed to be a rodent-specific phenomenon not occurring in humans. In the current study we performed a placebo-controlled, randomized cross-over trial and demonstrated with MRI measurements that one-week rifampicin treatment led to a small but statistically significant increase in liver volume-to-body weight ratio in humans. A limitation of this study was that MRI as a macroscopic method does not provide information on the cellular structure of the liver and therefore, we are not able to define cellular origin of the increased liver size nor to distinguish hypertrophy from hyperplasia. Compared to the effects of PXR ligands on rodent liver size, the observed rifampicin-induced increase in liver size in healthy volunteers is modest.

There is only one previous study with 6-day rifampicin dosing demonstrating an increase in liver volume (7.6% increase in median volume) in healthy volunteers as measured with hepatic magnetic resonance volumetry (Reichel et al. 1997). However, that one-arm trial was smaller (n=10) and did not employ a control arm. In aggregate, these findings suggest that PXR ligands may affect liver size in humans, and it remains possible that long-term treatment could have a stronger effect. Thus, PXR activation could be a useful mechanism to support liver regeneration in humans.

Many studies have reported that PXR activation induces steatosis in mouse liver and lipid accumulation has been observed also in human cell models (Bitter et al. 2015; Hakkola et al. 2016). In this study, we did not detect any effect on hepatic fat (triglyceride) content by rifampicin treatment. This suggests that there are either species differences in hepatic lipid metabolism in response to PXR activators in vivo, or the current study conditions did not favor hepatic lipid accumulation. Recently, we reported that 4-day PCN treatment of mice with diet-induced obesity dramatically promoted liver steatosis, while no liver steatosis was observed with similar PCN treatment in mice fed with normal chow or mice fed only short term with high-fat diet (Karpale et al. 2023). These results demonstrated that the metabolic state may affect metabolic consequences of PXR activation. Healthy, normal-weight volunteers enrolled in our study may be rather resistant to the metabolic effects of PXR activation and in theory individuals with existing metabolic disturbances, such as people with obesity, could be more susceptible to harmful metabolic effects. On the other hand, we have previously demonstrated that similar treatment with rifampicin impaired glucose tolerance in heathy volunteers (Rysä et al. 2013).

In the current study we describe a novel protocol to study mechanisms of PXR-mediated liver growth in mouse by re-expression of mPXR in the livers of *Pxr*^−/−^ mice. The results indicate that re-expression alone is not sufficient to induce liver growth, but combined with PCN treatment to activate mPXR, it induces rapid and strong liver hypertrophy. Interestingly, we observed a clear sex difference in PXR-mediated liver growth. Substantial liver enlargement was observed in the mPXR re-expressing male mice after 1-day PCN treatment, and this was further increased after 2-and 4-day treatment. However, in the female mice the mPXR re-expression and 4-day PCN treatment did not induce statistically significant increase in the liver weight, despite the tendency for it. In the mechanistic analysis, the livers of the female mice did show some activation of growth-related mechanisms, but systematically these responses were weaker than in the male mice. The observed differences between male and female mice could be attributed to structural and metabolic sexual dimorphism in the liver (Matz-Soja et al. 2026). These differences are regulated by multiple hormonal pathways, e.g., in males, pulsatile release of growth hormone drives the activation of signal transducer and activator transcription factor 5 (STAT5) signaling, which leads to more pronounced gluconeogenesis and β-oxidation in periportal liver zone. Contrastingly, in females, a continuous release of growth hormone activates STAT3, xenobiotic metabolism and lipogenesis in the pericentral liver zone (Matz-Soja et al. 2026). The observed sex difference in PXR-mediated liver growth appears to be opposite to closely related nuclear receptor CAR, since CAR ligand TCPOBOP was reported to induce stronger proliferative response in female mouse liver (Ledda-Columbano et al. 2003).

The observed liver growth in the mPXR expressing PCN treated mice involved both hepatocyte hypertrophy and hyperplasia. While the proliferation marker Ki-67 was strongly associated with the observed liver growth in the mPXR expressing PCN treated mice livers, we also observed a small number of proliferative cells in the control group, as well as in the animals, which did not receive PCN treatment. We hypothesize that this could be caused by adenovirus “leakiness”, where adenovirus transcription factor early region 2 and 4 (E2 and E4) associated genes are expressed in a E1A gene-independent manner. Machitani et al*.* have demonstrated that NF-kB can promote adenovirus leakiness in replication incompetent cells (Machitani et al. 2016). A fusion protein of open reading frames 6 and 7, a gene encoded by E4, has been shown to act as a viral transactivator of transcription factor E2F, which is involved in cell cycle progression (Täuber and Dobner 2001).

Several recent studies have investigated mechanisms of PXR-mediated liver growth and shown that PXR induces liver hypertrophy and promotes liver regeneration through YAP activation in mice (Abe et al. 2019; Jiang et al., S. Zhang et al. 2023). PXR has been shown to interact with YAP and to promote YAP nuclear localization, and on the other hand, YAP knockdown prevented PCN-mediated liver hypertrophy (Abe et al. 2019; Jiang et al. 2019). Therefore, rather compelling evidence indicates the crucial role of YAP in PXR-mediated liver growth. In the current study we did not observe clear evidence for activation of YAP by PXR activation despite the fact that MST1, a core component of the mammalian Hippo pathway along with YAP, was downregulated. In contrast, PCN promoted YAP phosphorylation, and the nuclear YAP level was unaltered at 1-day time point and decreased at 4-day time point after PCN treatment. Furthermore, the mRNA expression profile of the proliferation-related genes did not support the hypothesis on YAP-mediated regulation. Nevertheless, these results do not exclude the potential role of YAP in the early phase of liver growth before our first time point of 1 day.

Our results indicated strong activation of the AKT–MTOR-pathway in response to PXR activation. Both DigiWest screening and immunoblotting revealed increased phosphorylation of AKT and MTOR. The increased phosphorylation of the S6K1 downstream target S6 was especially strong. Along with the Hippo-pathway, the AKT–MTOR -pathway is one of the predominant pathways involved in organ size control and liver regeneration (Tumaneng et al. 2012; Ma et al. 2025) and deletion of the MTOR target protein S6K1 impaired liver proliferation after partial hepatectomy (Espeillac et al. 2011). Furthermore, the inhibition of eukaryotic translation initiation factor 4E-binding protein 1 (4EBP1) by MTOR allows protein translation and promotes proliferation (Tumaneng et al. 2012).

Additionally, we showed that the AKT target FOXO1 was inactivated by increased phosphorylation. This agrees with an earlier study reporting that AKT-mediated FOXO1 inhibition is required for liver regeneration (Pauta et al. 2016). Indeed, cell hypertrophy, proliferation, glycogenesis and lipid drop formation are all reduced by AKT-deficiency, and these effects are partly restored by FOXO1 deletion (Pauta et al. 2016). Interestingly, PXR is also known to inhibit FOXO1 by direct interaction (Kodama et al. 2004). PXR has been previously reported to promote hepatocyte proliferation also through inhibition of FOXO3 emphasizing the importance of the FOXO family in PXR-mediated liver growth (Shizu et al. 2016). There is also interplay between the Hippo and the AKT–MTOR pathways. Loss of MST1 leads to MTORC1 activation and, on the other hand, MST1 promotes FOXO1 nuclear translocation (Tumaneng et al. 2012). Thus, the MST1 downregulation observed in our study could play a role in the activation of the AKT–MTOR pathway.

Activation of re-expressed mPXR by PCN in the livers of male *Pxr*^*−/−*^ mice improved glucose tolerance in OGTT during the first 15 min in 1- and 2-day PCN-treated mice and at all the time points in 4-day treated male mice. OGTT AUC decreased significantly only after 4-day PCN treatment. In agreement with the lack of effect on liver size, there was no effect on glucose tolerance in female mice or in the male mice with mPXR re-expression alone without the ligand treatment. This suggests that the observed changes in glucose tolerance are associated with liver growth. FOXO1 plays an important role in glucose metabolism by promoting gluconeogenetic enzymes. In agreement with FOXO1 inhibition, we observed repression of *Pck1* in response to PCN treatment. Inhibition of gluconeogenesis could play some role in the observed reduced blood glucose during the OGTT. However, there is probably an increase in glucose disposal to explain the strong effect on glucose tolerance after 4-day PCN treatment. Indeed, it could be hypothesized that this is due to greater energy demand for the growing, enlarged livers. *Gck* and *Glut1* mRNA levels were decreased and the expression of the major hepatic glucose transporter *Glut2* was not affected in response to the PCN in mPXR expressing mouse livers. However, as intracellular glucose (via acetyl-CoA and further citrate) is most likely utilized, through a PXR-mediated mechanism (Hakkola et al. 2016) for the synthesis of fatty acids for the formation of plasma membranes and cell organelles of the new and hypertrophying hepatocytes, the induction of glucose transporters is not required for the increase in transmembrane glucose influx. From an evolutionary perspective, it is logical that the PXR-mediated pathway, sensing the potentially hepatotoxic chemicals entering the body, 1) regulates the enzymes and transporters responsible for hepatic detoxification, 2) controls the liver regeneration machinery, and 3) ensures the energy needs of these processes.

In summary, we discovered that one-week rifampicin treatment leads to a small but significant increase in the liver volume-to-body weight ratio in humans suggesting liver growth-promoting effects by PXR activation also in humans. Furthermore, we described a novel protocol to study mechanisms of PXR-mediated liver growth in mice and with this protocol identified the AKT–MTOR pathway to be strongly activated in the in the PXR-mediated liver enlargement. We also described a major sex difference in PXR-mediated liver growth. Uncovering the pathways and functions associated with PXR-dependent liver growth could potentially lead to targeting these mechanisms to bolster liver regeneration after partial hepatectomy in patients with liver tumor, or in liver injury.

## Supplementary Information

Below is the link to the electronic supplementary material.

Supplementary Material 1

Supplementary Material 2

## Data Availability

RNA-sequencing data were deposited into the Gene Expression Omnibus database under accession numbers GSE297681 and GSE297893. All other data supporting the findings of this study are available within the paper and its Supplementary Information.

## Abbreviations

4E-BP1: Eukaryotic translation initiation factor 4E-binding protein 1
4βHC: 4β-Hydroxycholesterol
Ad: Adenovirus
AKT: Protein kinase B
AUC: Area under curve
BMI: Body-mass index
BW: Body weight
CAR: Constitutive androstane receptor
Ccnd1: Cyclin D1
Ccne1: Cyclin E4
Cd163: Cluster of differentiation 163
Cdk4: Cyclin-dependent kinase 4
Ctgf: Connective tissue growth factor
CYP2A: Cytochrome p450 2A
Cyp2c55: Cytochrome p450 2c55
CYP3A4: Cytochrome P450 3A
Cyr61: Cysteine-rich angiogenic inducer 61
d: Days
DEGs: Differentially expressed sequences
EIF-4E: Eukaryotic translation initiation factor 4E
F: Females
FOXO1: Forkhead box protein O1
Gapdh: Glyceraldehyde 3-phosphate dehydrogenase
Gbp2: Interferon-induced guanylate-binding protein 2
Gbp7: Interferon-induced guanylate-binding protein 7
Gck: Glucokinase
GFP: Green fluorescent protein
Glut 2: Glucose transporter 2
Glut1: Glucose transporter 1
GSK3: Glycogen synthase kinase 3
Gstm3: Glutathione S-transferase mu 3
ID1: Inhibitor of differentiation
Igtb: Interferon gamma induced GTPase
Iigp1: Interferon inducible GTPase 1
IPA: Ingenuity pathway analysis
IVC: Individually ventilated cages
LATS1: Large tumor suppressor kinase 1
LC–MS: Liquid chromatography–mass spectrometry
LV: Liver volume
LV/BW: Liver volume-to-body weight ratio
LW: Liver weight
LXR: Liver X receptor
M: Males
Mdm2: Mouse double minute 2 homolog
MOB1: MOB kinase activator 1A
mPXR: Murine PXR
MSP: Macrophage-stimulating protein
MST1: Macrophage stimulating 1
MTOR: Mammalian target of rapamycin
MYC: MYC proto-oncogene
NO PCN: Mice without PCN treatment
OGTT: Oral glucose tolerance test
p: Phospho
p21: Cyclin dependent kinase inhibitor 1
p53: Tumor protein p53
Pai1: Plasminogen activator inhibitor-1
Pck1: Phosphoenolpyruvate carboxykinase 1
PCN: Pregnenolone−16α-carbonitrile
PI3K: Phosphoinositide 3-kinase
PPARα: Peroxisome proliferator receptor α
PXR: Pregnane X receptor
PXR^−/−^: PXR knockout
qPCR: Quantitative polymerase chain reaction
RON: Recepteur d'origine nantais
S6: Ribosomal protein S6
S6K1: Ribosomal protein S6 kinase beta-1
Sult2a2: Sulfotransferase 2a2
Sult2a5: Sulfotransferase 2a5
TAZ: Transcriptional coactivator with PDZ-binding motif
Tbp: TATA-binding protein
TEAD: TEA domain family member 1
Tgtp1: T-cell specific GTPase 1
WNT3: Proto-oncogene protein Wnt-3
YAP: Yes-associated protein
