# Supplementary material for: Pregnane X receptor-mediated liver growth: a controlled clinical trial in healthy volunteers and identification of the role of AKT–MTOR pathway in mouse
Source: Arch Toxicol. 2026 Aug 26;100(12):5513–28. doi: 10.1007/s00204-026-04526-5 (PMC13748098; doi:10.1007/s00204-026-04526-5)
Supplement: Supplementary file 1 [file 204_2026_4526_MOESM1_ESM.pdf]

A

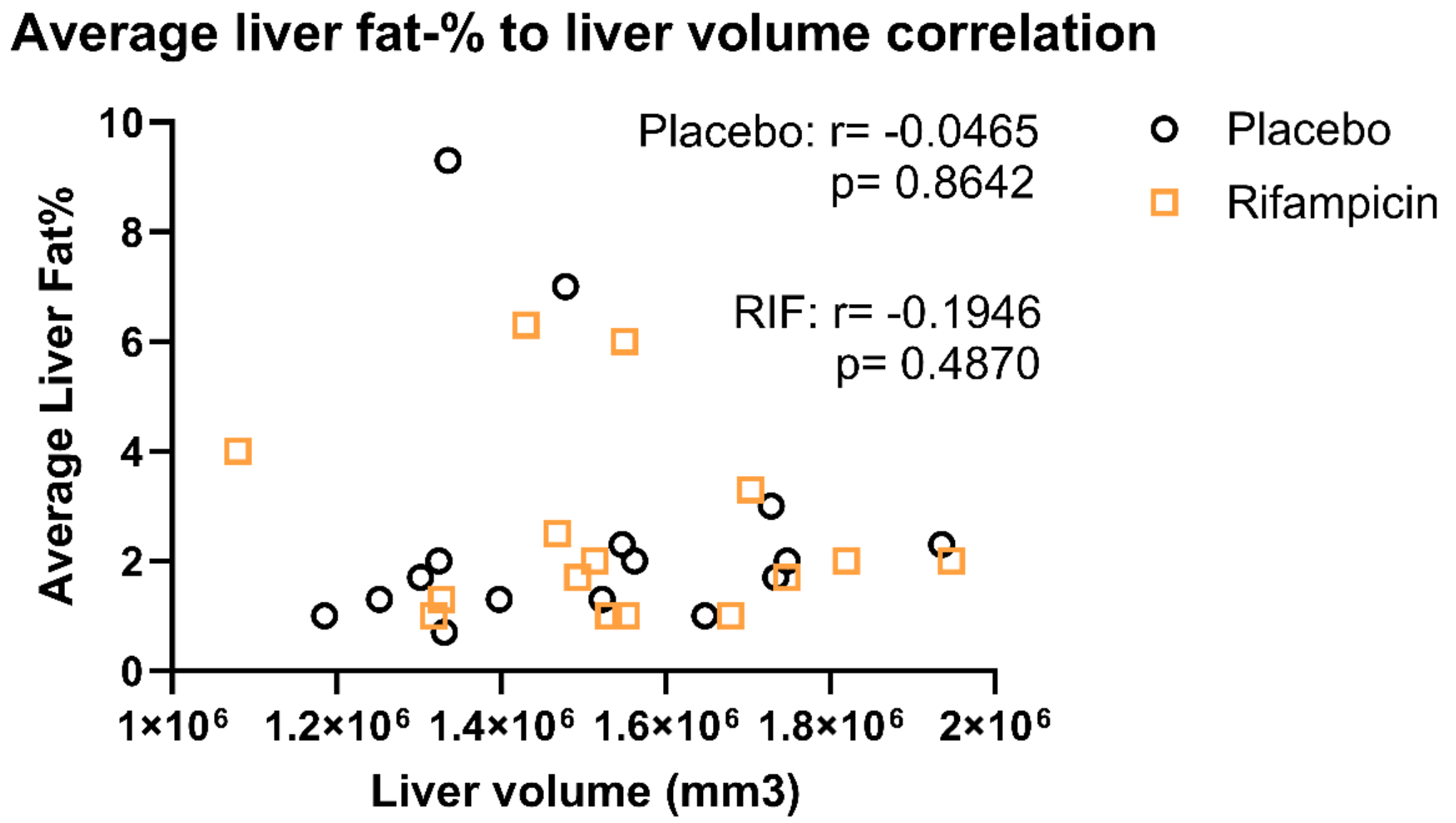

B

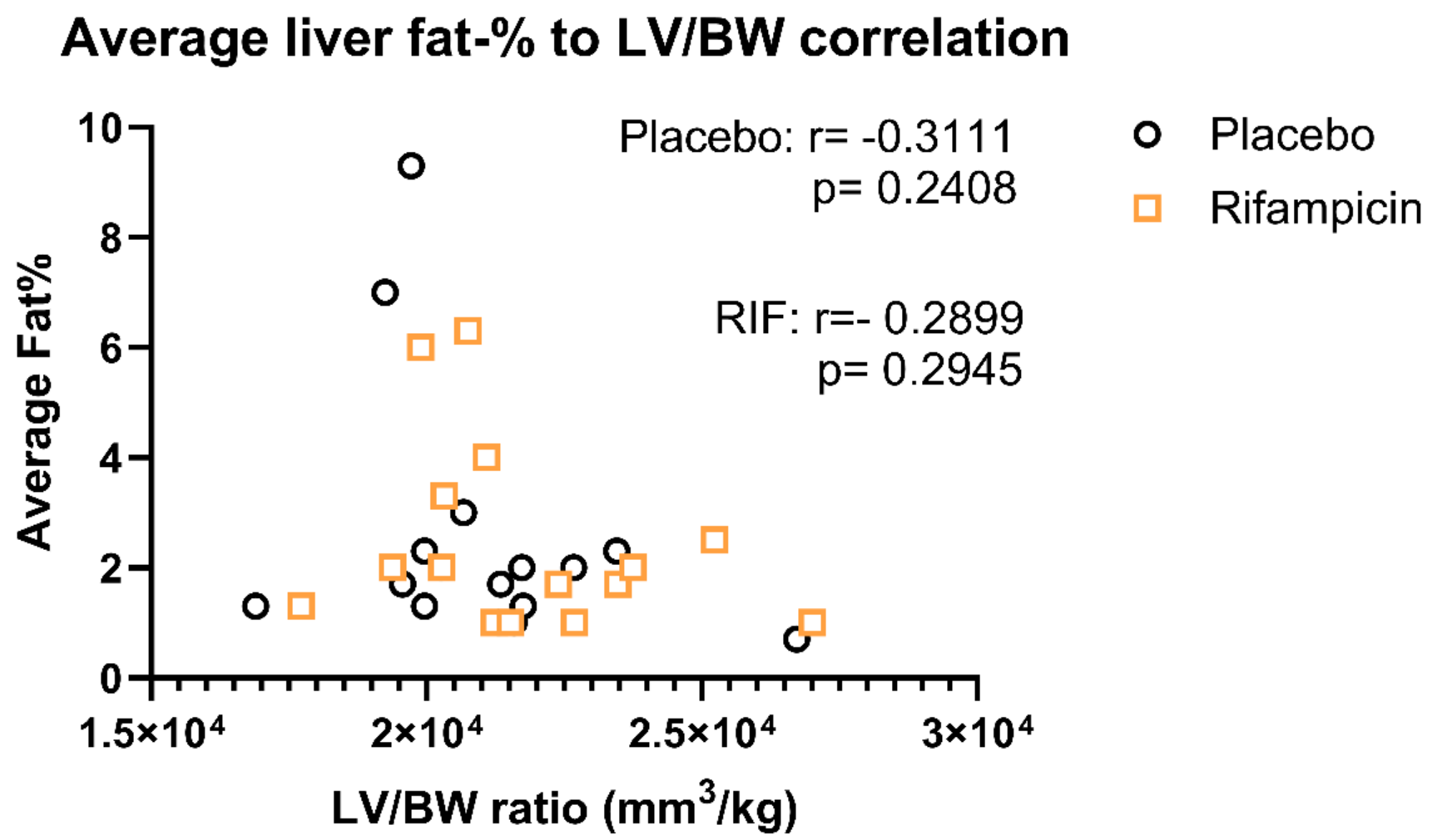

Supplementary Figure 1. Rifa-Stea clinical trial. Correlation graphs of A) average liver fat-% to liver volume and B) average liver fat-% to liver volume-to-body weight ratio (LV/BW). No significant correlation can be found in either rifampicin (RIF) or placebo group, *p<0.05.

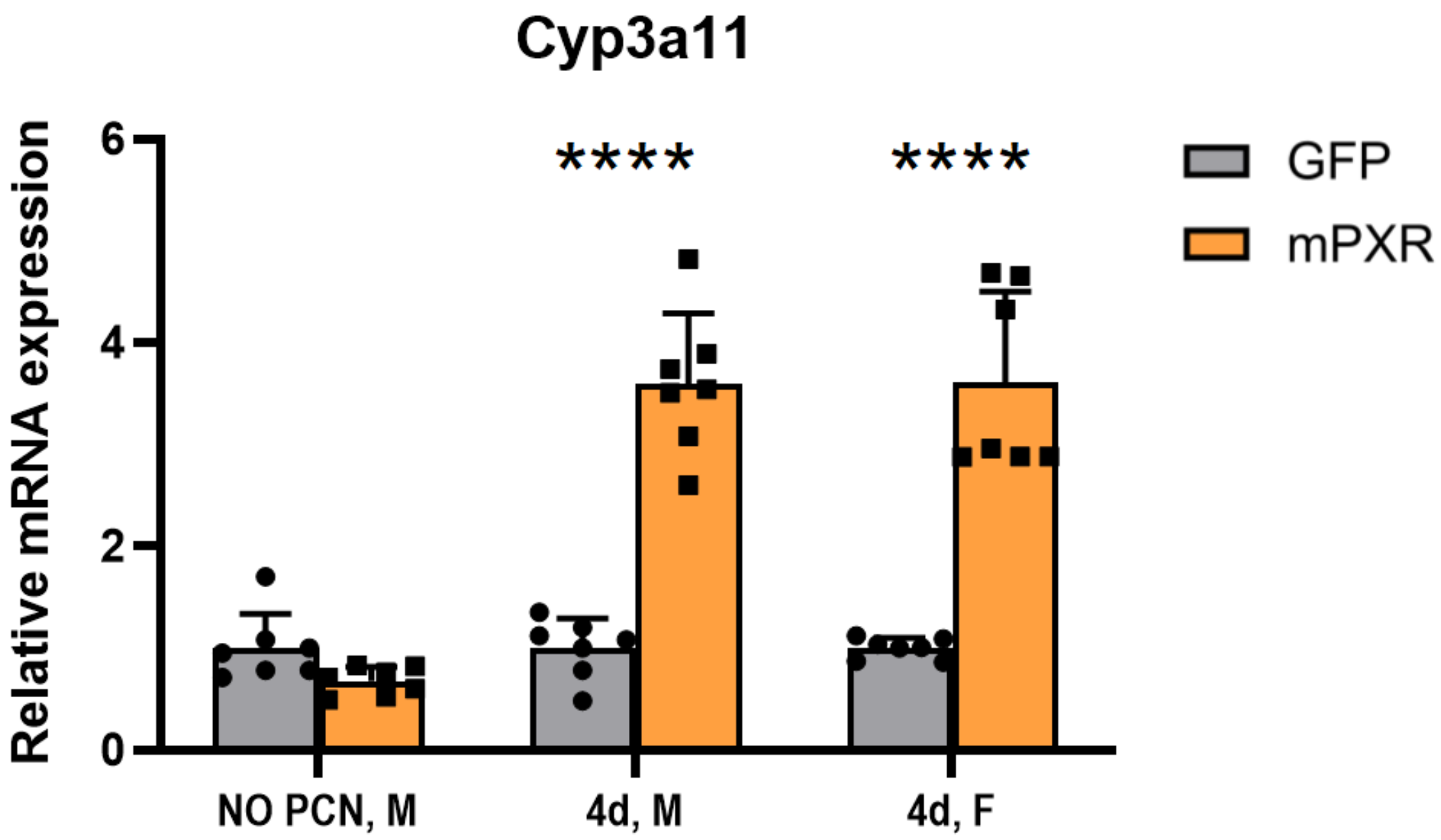

Supplementary Figure 2. Effect of 4-day PCN treatment on hepatic *Cyp3a11* mRNA level in mPXR re-expressing male and female mice compared to control GFP-groups. No change in *Cyp3a11* expression was observed in the mPXR re-expressing male mice without PCN treatment (NO PCN). M=males, F=females, d=days. Results are presented as mean ± SD (n=7). ****p<0.0001.

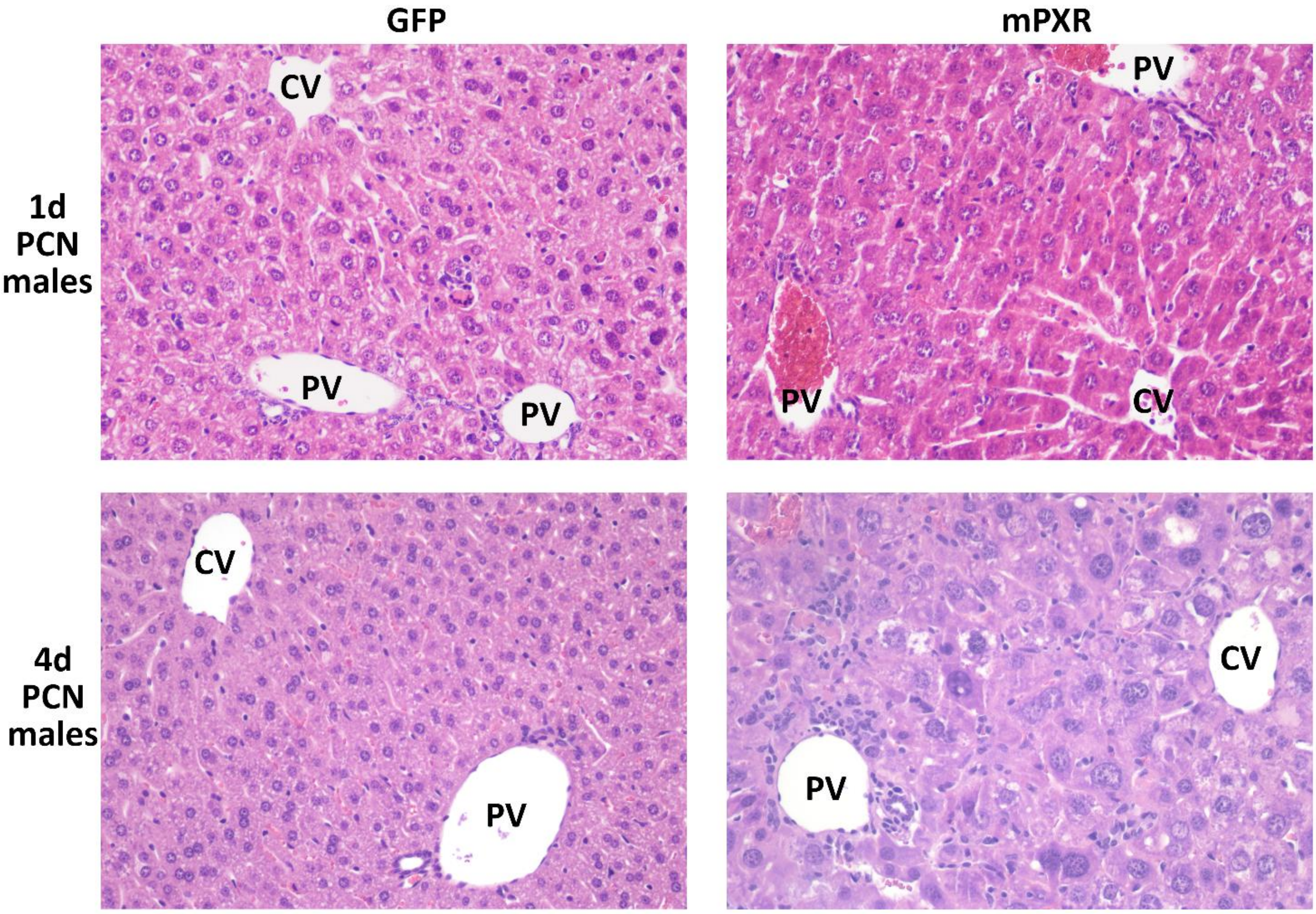

Supplementary Figure 3. HE-staining of the livers from 1- and 4-day PCN-treated male mice. Image taken at 20x magnification. CV=central vein, PV=portal vein.

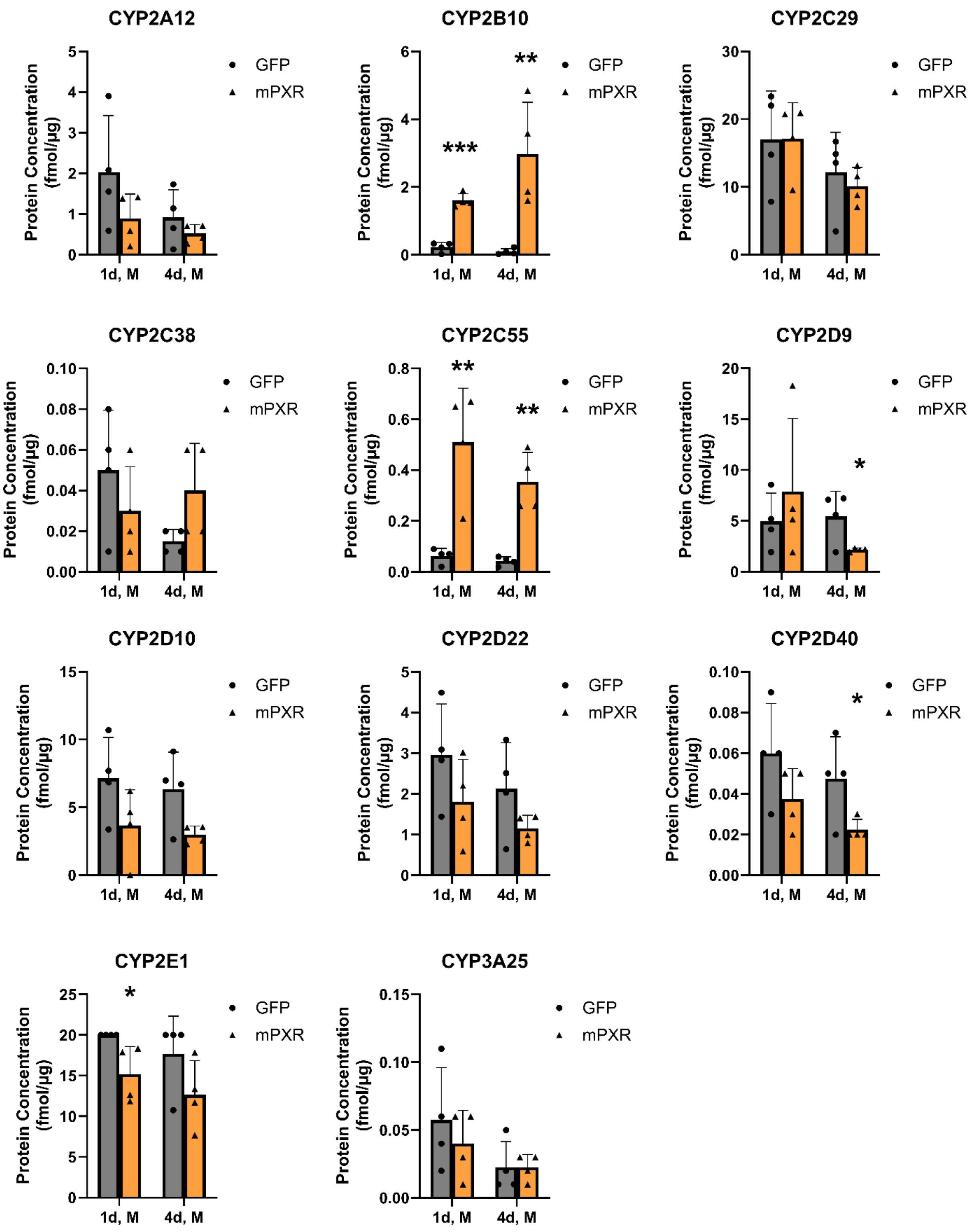

Supplementary Figure 4. Immunoaffinity-LC-MS/MS analysis of CYP proteins from the livers of 1- and 4-day PCN-treated male mice. In CYPE1 panel several values exceed the upper detection limit of the machine (>20 fmol/µg) and have therefore been marked with value of 20. M=males, d=days. Results are presented as mean ± SD (n=4). *p<0.05; **p<0.01.

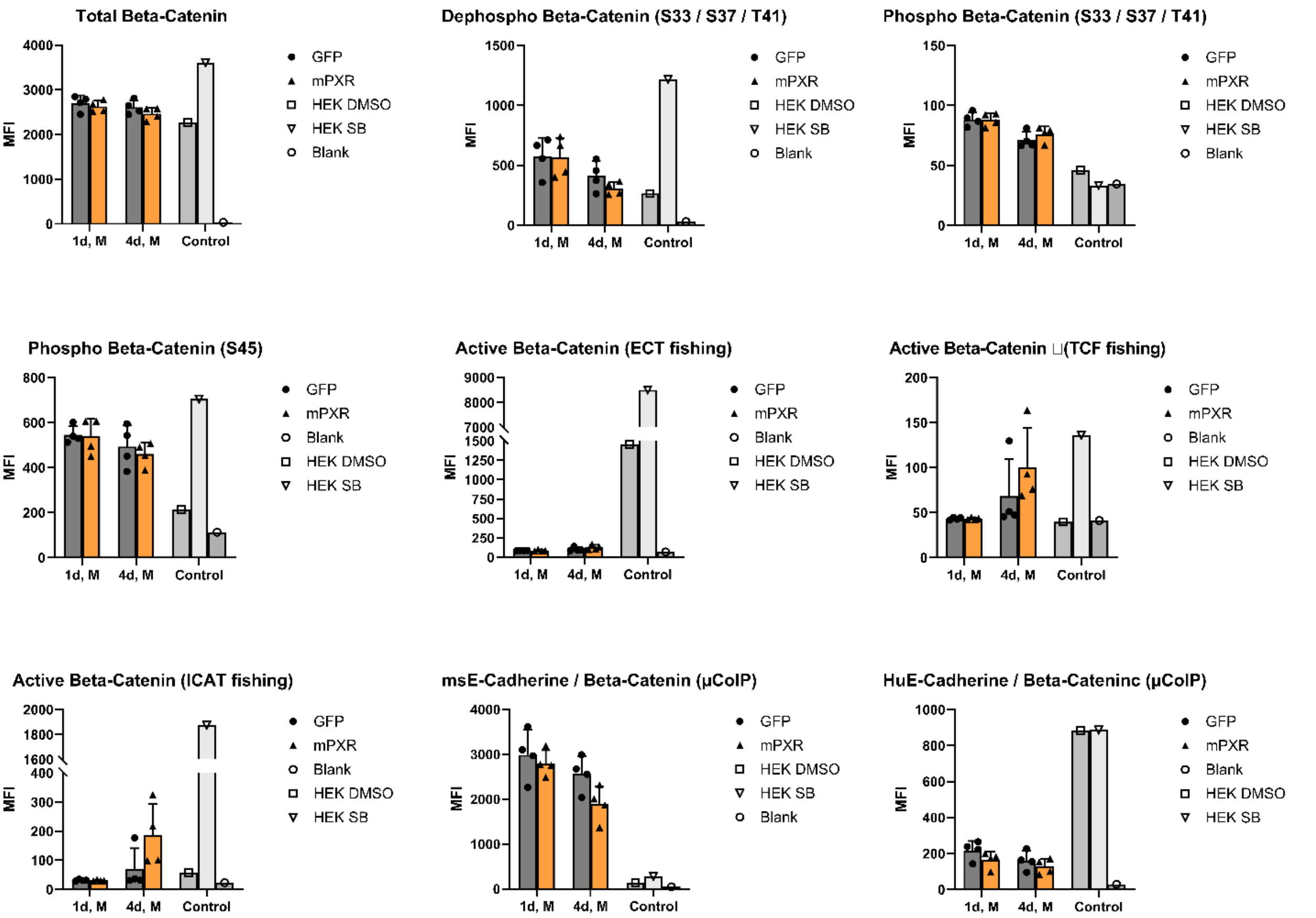

Supplementary Figure 5. Microsphere-based array panel for Beta-catenin profiling from 1- and 4-day PCN-treated mice livers. There are no statistically significant differences between the groups. MFI=median fluorescence intensity, M=males, d=days. Results are presented as mean ± SD (n=4 for GFP and mPXR, and n=1 for controls).

HIPPO-YAP SIGNALING 1 day males

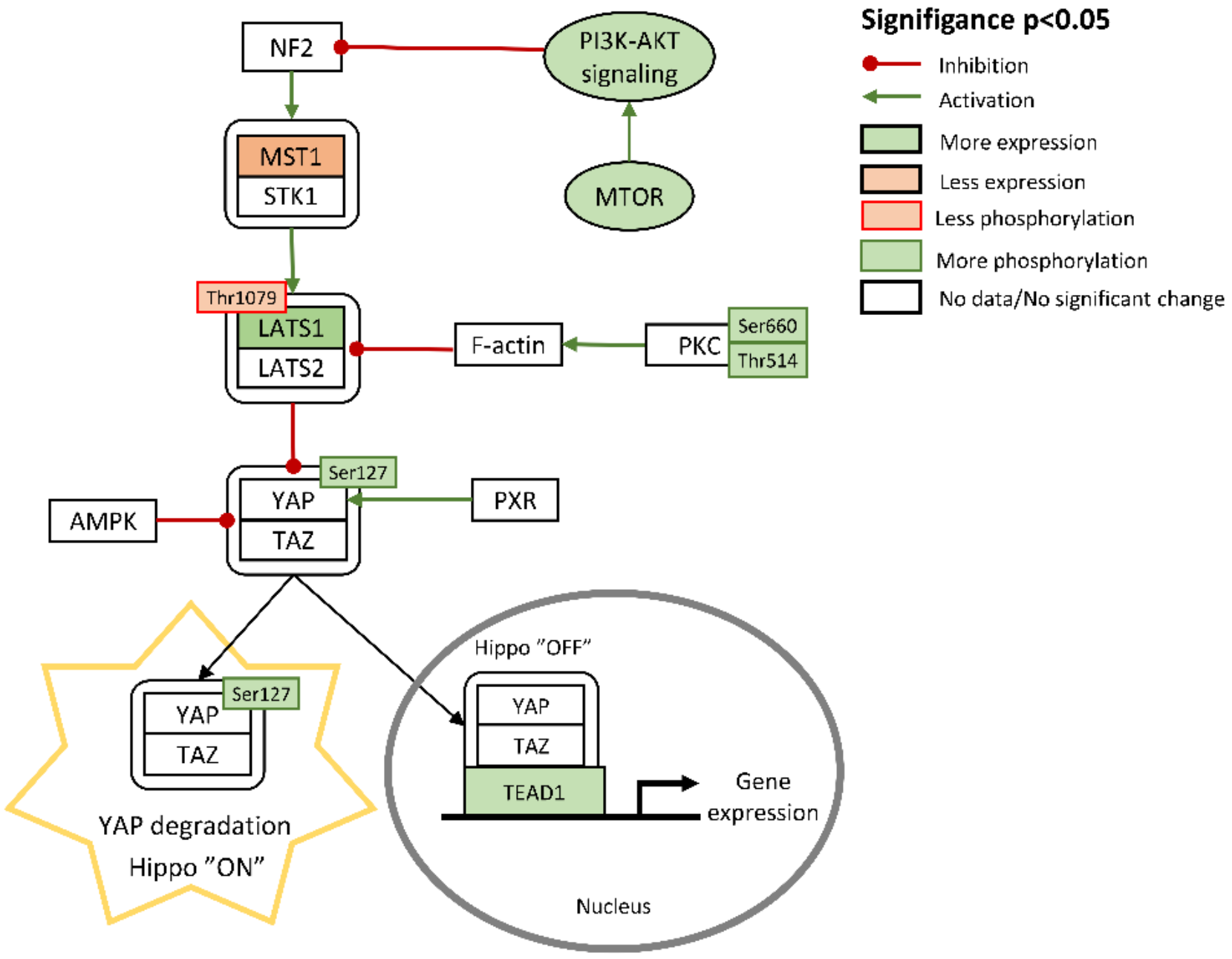

HIPPO-YAP SIGNALING 4 day males

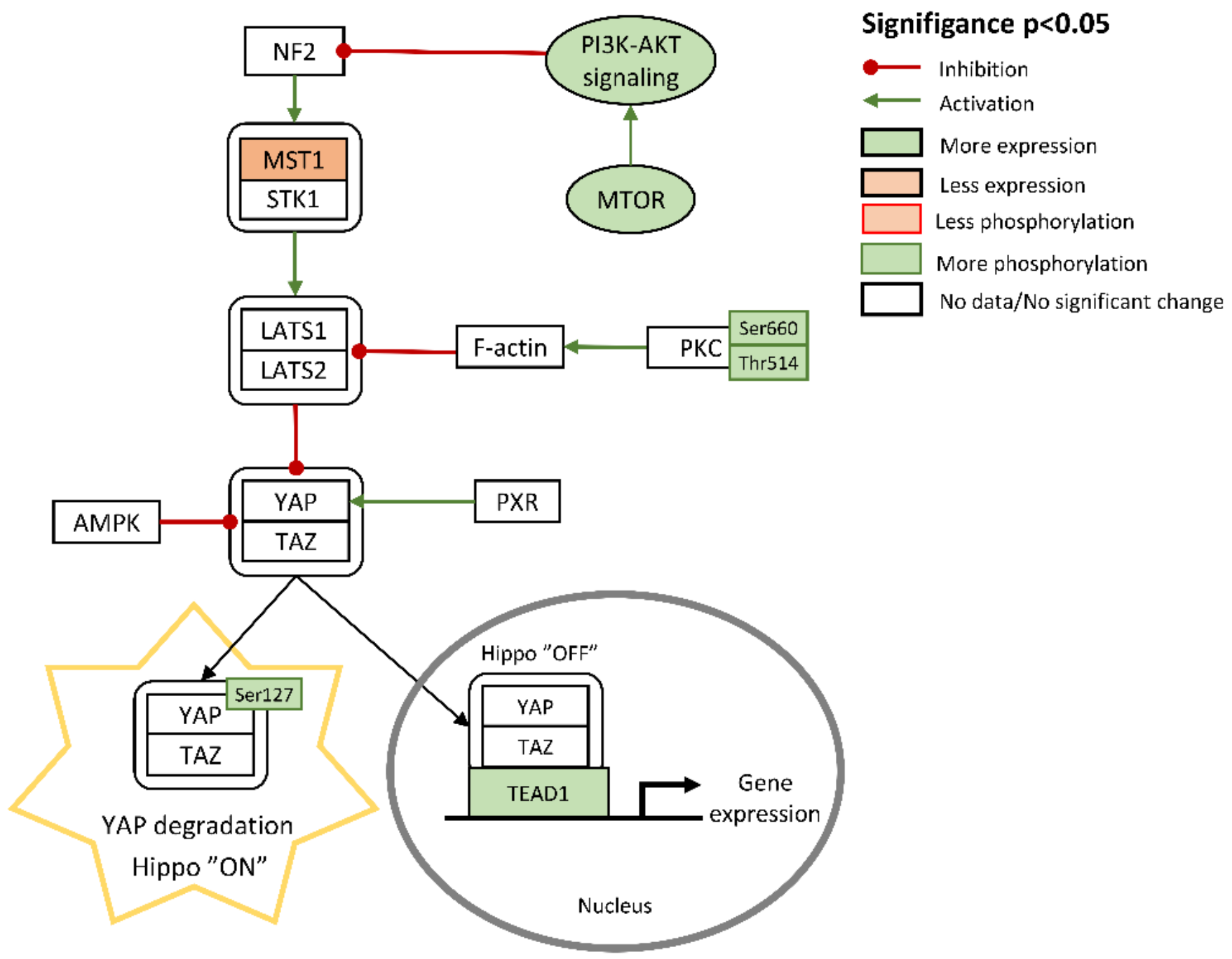

Supplementary Figure 6. Graphical representation of the effect of mPXR activation on HIPPO-YAP pathway in 1- and 4-day PCN-treated male mice. Statistical significance level $p<0.05$.

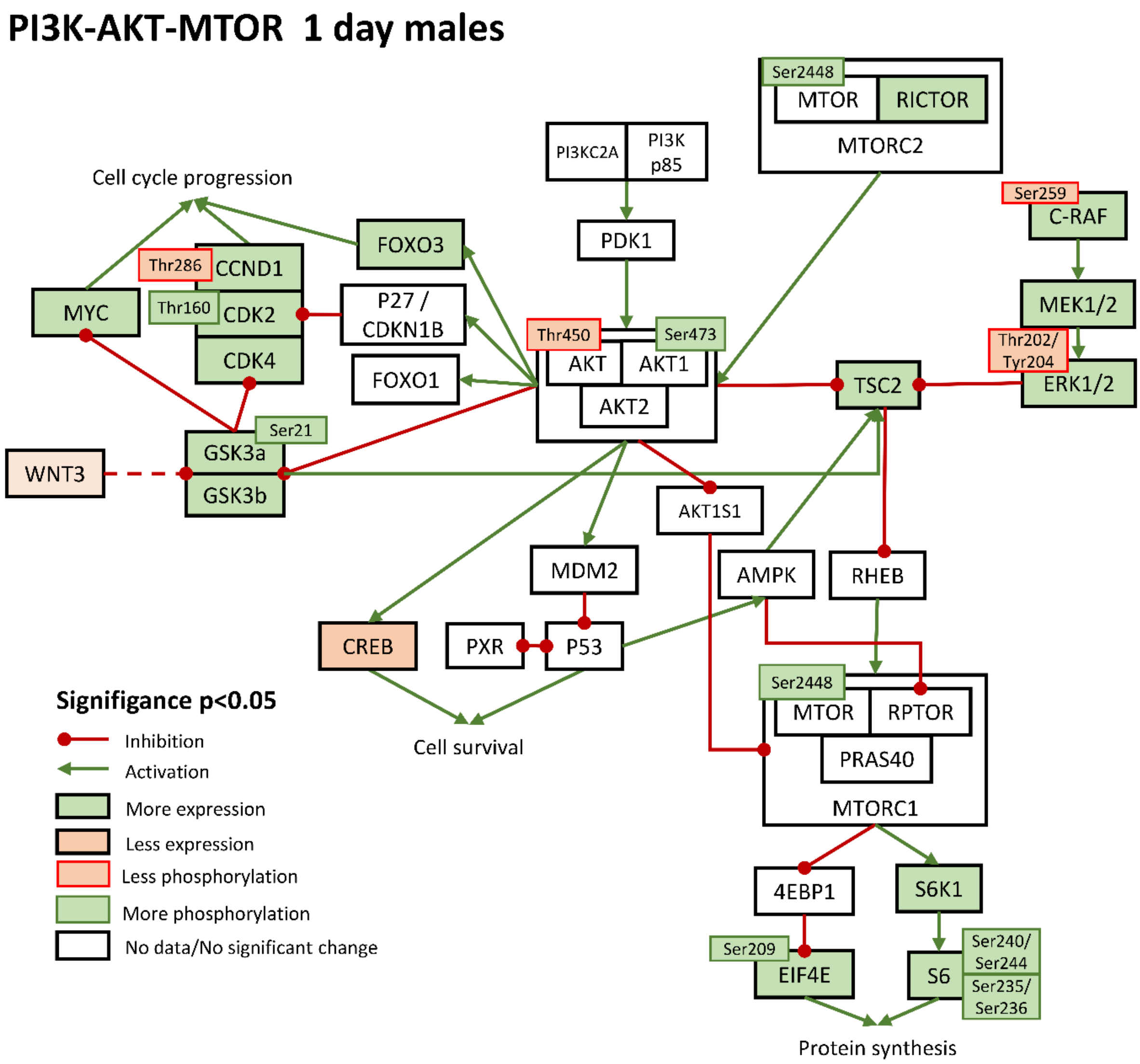

Supplementary Figure 7. Graphical representation of the effect of mPXR activation on PI3K-AKT-MTOR-pathway in 1-day PCN-treated male mice. Statistical significance level $p<0.05$.
